# Assessing associations between individual-level social determinants of health and COVID-19 hospitalizations: Investigating racial/ethnic disparities among people living with human immunodeficiency virus (HIV) in the U.S. National COVID Cohort Collaborative (N3C)

**DOI:** 10.1017/cts.2024.550

**Published:** 2024-05-21

**Authors:** Dimple Vaidya, Kenneth J. Wilkins, Eric Hurwitz, Jessica Y. Islam, Dongmei Li, Jing Sun, Sandra E. Safo, Jennifer M. Ross, Shukri Hassan, Elaine Hill, Bohdan Nosyk, Cara D. Varley, Nada Fadul, Marlene Camacho-Rivera, Charisse Madlock-Brown, Rena C. Patel

**Affiliations:** 1 Departments of Medicine and Epidemiology, University of Washington, Seattle, WA, USA; 2 Biostatistics Program, Office of the Director, National Institute of Diabetes and Digestive and Kidney Diseases, National Institutes of Health, Bethesda, MD, USA; 3 Department of Biomedical Informatics, University of Colorado Anschutz Medical Campus, Aurora, CO, USA; 4 Virginia Commonwealth University, Richmond, VA, USA; 5 Department of Cancer Epidemiology, H. Lee Moffitt Cancer Center and Research Institute, Tampa, FL, USA; 6 Department of Oncologic Sciences, University of South Florida, Tampa, FL, USA; 7 Department of Clinical and Translational Research, University of Rochester Medical Center, Rochester, NY, USA; 8 Department of Epidemiology, Johns Hopkins Bloomberg School of Public Health, Baltimore, MD, USA; 9 Department of Biostatistics, School of Public Health, University of Minnesota, Minneapolis, MN, USA; 10 Department of Medicine, University of Washington, Seattle, WA, USA; 11 Department of Public Health Sciences, University of Rochester Medical Center, Rochester, NY, USA; 12 Faculty of Health Sciences, Simon Fraser University, Burnaby, BC, Canada; 13 Oregon Health & Science University, School of Medicine; Oregon Health & Science University-Portland State University School of Public Health, Portland, OR, USA; 14 Department of Medicine, University of Nebraska Medical Center, Omaha, NE, USA; 15 Department of Community Health Sciences, SUNY Downstate School of Public Health, Brooklyn, NY, USA; 16 Acute and Critical Care Division, College of Nursing, University of Iowa, Iowa City, IA, USA; 17 Department of Medicine, University of Alabama at Birmingham, Birmingham, AL, USA

**Keywords:** Individual-level, social determinants of health, human immunodeficiency virus, COVID-19, race/ethnicity

## Abstract

**Background:**

Leveraging the National COVID-19 Cohort Collaborative (N3C), a nationally sampled electronic health records repository, we explored associations between individual-level social determinants of health (SDoH) and COVID-19-related hospitalizations among racialized minority people with human immunodeficiency virus (HIV) (PWH), who have been historically adversely affected by SDoH.

**Methods:**

We retrospectively studied PWH and people without HIV (PWoH) using N3C data from January 2020 to November 2023. We evaluated SDoH variables across three domains in the Healthy People 2030 framework: (1) healthcare access, (2) economic stability, and (3) social cohesion with our primary outcome, COVID-19-related hospitalization. We conducted hierarchically nested additive and adjusted mixed-effects logistic regression models, stratifying by HIV status and race/ethnicity groups, accounting for age, sex, comorbidities, and data partners.

**Results:**

Our analytic sample included 280,441 individuals from 24 data partner sites, where 3,291 (1.17%) were PWH, with racialized minority PWH having higher proportions of adverse SDoH exposures than racialized minority PWoH. COVID-19-related hospitalizations occurred in 11.23% of all individuals (9.17% among PWH, 11.26% among PWoH). In our initial additive modeling, we observed that all three SDoH domains were significantly associated with hospitalizations, even with progressive adjustments (adjusted odds ratios [aOR] range 1.36–1.97). Subsequently, our HIV-stratified analyses indicated economic instability was associated with hospitalization in both PWH and PWoH (aOR range 1.35–1.48). Lastly, our fully adjusted, race/ethnicity-stratified analysis, indicated access to healthcare issues was associated with hospitalization across various racialized groups (aOR range 1.36–2.00).

**Conclusion:**

Our study underscores the importance of assessing individual-level SDoH variables to unravel the complex interplay of these factors for racialized minority groups.

## Introduction

The COVID-19 pandemic has disproportionately affected minority communities in the United States (U.S.) and exposed long-standing health-care disparities evident in other diseases, such as the human immunodeficiency virus (HIV) epidemic. While in the U.S. alone, the number of COVID-19 cases has surpassed 100 million, with more than 1.1 million deaths [1], both direct impacts of the infections and subsequent adverse outcomes, as well as indirect impacts of social and economic consequences of shutdowns, stay-at-home orders, and more, have differentially affected persons of racial/ethnic minority, hereafter termed “racialized minority,” communities[2]. U.S. Black/African American persons have experienced a higher incidence of COVID-19, along with increased hospitalization, intensive care unit admissions, and mortality rates compared to the non-Hispanic/Latinx White persons [3]. These inequities likely stem from both health-related and social and structural risk factors. Racialized minority groups face higher rates of chronic illnesses and limited healthcare access, compounded by living and working conditions that increase social vulnerability to severe COVID-19 outcomes [3,4]. Therefore, a deeper understanding of structural vulnerability risk factors contributing to adverse outcomes in racialized minority communities is critical.

According to Healthy People 2030, the social determinants, sometimes also termed drivers, of health (SDoH) encompass various conditions in which individuals are born, grow, develop, work, play, worship, and age, significantly influencing an array of health outcomes, functionality, and risks to quality of life [5]. SDoH factors, such as education and early childhood development, urban planning and community development, housing, and employment, often account for disparities in health outcomes and contribute to health inequities [6]. Concurrently, heterogeneity in COVID-19 risk exists due to area-level factors like housing density, occupation, and structural racism at population levels [7]. These multi-level social dynamics contribute to the emergence of risk factors influencing COVID-19 exposure and susceptibility, as well as differences in treatment-seeking behavior and access to care. Consequently, the pandemic’s social, psychological, health, and economic consequences disproportionately affect individuals’ health and well-being based on their overall social vulnerability [8].

Just like in the COVID-19 pandemic, racialized minority communities have historically faced marked racial/ethnic inequities in the U.S. HIV epidemic. Among people with HIV (PWH), for example, Black/African American and Latinx/Hispanic PWH share a higher burden of the HIV epidemic compared to their White counterparts. Although Black/African Americans constituted only 12% of the U.S. population, Black/African Americans accounted for 42% of HIV-related mortality in 2021 [9]. SDoH factors likely form the foundation for these health disparities [10]. Racialized minority communities face not only inequitable exposure due to social vulnerability but also a higher prevalence of preexisting chronic conditions like HIV. These conditions likely heighten the risk of adverse outcomes from COVID-19, as we have previously shown that racialized minority PWH experienced a disproportionate burden of COVID-19 infections and severity compared to racialized minority people without HIV (PWoH) or White PWH [11]. Given the disproportionate impact of the COVID-19 pandemic on PWH, it is crucial to explore the significance of SDoH to understand potential pathways to mitigate disparities among racial/ethnic minorities affected by HIV.

Furthermore, SDoH factors are often measured at area-level exposures, such as at census tract levels, zip codes, or counties [12]. These area-level SDoH exposures offer critical insights into contextual factors that may be driving underlying individual access to healthcare, transportation, food, and more. However, aggregation at the area-level exposures often loses the granularity or heterogeneity of the subpopulations residing within specific areas. Given the glaring impact of SDoH on COVID-19-related outcomes, there has been growing interest in ascertaining and using individual-level SDoH data. Studies with individual-level SDoH data with national sampling for COVID-19 are scarce [13] and, within the overlap of HIV and COVID-19, are non-existent. Therefore, a critical gap remains in understanding the impact of individual-level SDoH at the intersections of COVID-19, HIV, and race/ethnicity, specifically in understanding how individual SDoH contributes to the severity of COVID-19 among PWH. Given this context, our research seeks to address three primary questions:

- Are individual-level SDoH, within (1) healthcare access, (2) economic stability, and (3) social cohesion domains, associated with COVID-19-related hospitalization, and do these associations persist after adjustments for demographic and baseline health covariates?
- How do the observed associations differ between PWH and PWoH?
- In what ways can the joint contributions of SDoH and HIV status vary among racialized minority communities concerning the outcome of COVID-19-related hospitalization?

By investigating these questions, our research aims to fill the crucial gap in understanding how individual-level SDoH variables influence COVID-19 outcomes in PWH and PWoH using a data-driven approach. This will contribute to the existing body of knowledge and inform targeted interventions and policy decisions aimed at reducing the impact of COVID-19 among this vulnerable population.

## Methods

### Overall Structure, Data Sources, and Study Population

We used data from the National COVID-19 Cohort Collaborative (N3C) Enclave sponsored by the U.S. National Institutes of Health (NIH) [14]. This data enclave includes harmonized de-identified clinical data on over 21 million individuals, including over 8.5 million COVID-19-positive individuals, across 80+ data partner sites from the U.S. Data partner sites contribute demographic, visit, vital status, medication, laboratory, diagnoses, and radiography data, with “look back” data back to January 2018 at their site, to a central data repository that is harmonized on a regular basis according to a common data model.

The N3C cohort includes COVID-19-positive individuals matched with two COVID-19-negative controls based on up to four sociodemographic variables (age, sex, race, and ethnicity) whenever available by data partner site. In this analysis, COVID-19 positivity is defined by: 1) a set of a priori-defined SARS-CoV-2 laboratory tests (that includes polymerase chain reaction (PCR) or antigen positivity, but not antibody positivity) or 2) a “strong positive” diagnostic code, with this cohort code available on GitHub [15]; our study utilized N3C Data Release-v148-2023-11-02 with Level 3 access granted.

### Ethical reviews

The N3C data transfer to the NIH is performed under a Johns Hopkins University Reliance Protocol (IRB00249128) or individual site agreements with NIH. The N3C data Enclave is approved through the NIH Institutional Review Board (IRB) and each investigator accessing the Enclave receives institutional IRB from their respective institution.

### Study design and analytic Sample

We conducted a retrospective cohort study using real-time electronic health record (EHR) data collected from January 1, 2020, through November 2, 2023. We included data from the first COVID-19 infection recorded for each person in N3C.

We identified PWH within the N3C Enclave using various Observational Medical Outcomes Partnership concepts, such as HIV diagnosis (ICD-10, SNOMED), relevant medications (RxNorm), and specific laboratory measurements (LOINC) [16]. Individuals using pre-exposure prophylaxis (PrEP), solely living with hepatitis B virus (HBV) infection but receiving HIV-related medications for HBV treatment or undergoing post-exposure prophylaxis were excluded from the PWH cohort. For this analysis, we opted to include PWH classified at our two highest confidence levels. We detail these confidence levels further in the Supplementary Text. Individuals not meeting our phenotyping criteria for HIV were considered PWoH.

### Outcome

We defined COVID-19-related hospitalization as a binary outcome, considering whether the patient was admitted to the hospital from the day before up to 16 days following the initial COVID-19 diagnosis [14]. This timeframe aligns with the periods specified by the Centers for Disease Control and Prevention [17].

### Exposures

We ascertained individual-level SDoH in N3C based on mapping to questions that appeared in the Epic® EHR SDoH Module at some sites [18,19]. The N3C data harmonization team mapped the SDoH questions via LOINC for ingestion into the system across five domains including food insecurity, transportation, financial strain, social connectedness, and stress categories. Sites that did not have an Epic SDoH Module but were still collecting information in a module question set had their data harmonized as above. Each question was further aligned with its respective Healthy People 2030 domains (i.e., (1) healthcare access & quality, (2) economic stability, (3) social & community context, (4) education access & quality, and (5) neighborhood & built environment), with the exception of stress, as it falls outside this framework [5]. Moreover, to ensure a comprehensive representation, SDoH experts reviewed and categorized each response, whether a binary yes/no vs. on a Likert scale, based on whether it pertained to a social need, risk, or instability (i.e., a positive response indicated some social vulnerability). When individuals had more than one question asked within one of the five Healthy People 2030 domains, we allowed any positive response within the domain to indicate social vulnerability. All individual-level SDoH data were ascertained prior to and up to 30 days after the first incident of COVID-19 infection to capture the most comprehensive and relevant SDoH information. When multiple data were available over more than one-time point, we selected the most recent SDoH data prior to the first incident of COVID-19 infection. Of note, in our analytic sample, only data for (1) healthcare access & quality, (2) economic stability, and (3) social & community context were available. The individual-level SDoH questions, responses, our categorization for Health People 2030 SDoH domains, and study metadata are available in Supplementary Table 1.

While responses regarding stress are sometimes included in the Epic® EHR SDoH Module, for this analysis, we chose to remove responses related to stress for two main reasons. First, we note that in conceptualization of stress in this analysis, stress likely falls within the pathways between other SDoH domains, HIV, and COVID-19 outcomes. We note that stress is not included as a domain within our chosen *a priori* SDoH framework, the Healthy People 2023, where it is considered an outcome of adverse SDoH [20]. Additionally, stress is also challenging to model. For example, research on the impact of stress on maternal health outcomes has found inconsistent results, in part due to how stress is measured [21]. Researchers did find that analyzing a combination of different kinds of stressors (e.g., environmental, access to care) provided a more consistent picture of maternal health outcomes [22]. Hence, incorporating stress into our framework poses challenges that necessitate a distinct and separate analysis.

Race/ethnicity was defined for each individual by combining race and ethnicity variables available in N3C Enclave. Individuals were classified as either: non-Hispanic (NH)-American Indian or Alaskan Native (AIAN), NH-Asian American, Native Hawaiian, or Pacific Islander (AANHPI), NH-Black/African American, Hispanic/Latinx of any race, and NH-White. Notably, our stratified model findings by race/ethnicity may have suppressed results for NH-AIAN individuals, due to small cell counts that result from cross-classifying the outcome with each unique combination of covariate values, if we had not employed model selection steps outlined in the Supplementary Text. However, we include findings per recommendations for reporting health research for this population [23]. Those with unknown, missing, and other NH race/ethnicity were excluded from analysis, due to both small cell counts and lack of interpretability.

### Covariates

We included the covariates of age, sex, and clinical comorbidity burden in the analysis due to their known associations with COVID-19 outcomes and data availability and quality within N3C [24,25]. We determined the age of each individual at the time of the first incident of COVID-19 infection. Age was then categorized into three categories: <45, 45–64, ≥65 years. Sex is represented as biological sex at birth. We assessed the clinical comorbidity burden via a modified Charlson Comorbidity Index (CCI) score, calculated using a combination of binary flags for comorbidities prior to each individual’s first incident COVID-19 infection date and excluded HIV, where comorbidities have been phenotyped and harmonized using N3C-vetted and -recommended concept sets. The weights for calculating CCI score follow the same definition as described in Charlson *et al* [26]. While a plethora of data exists regarding clinical factors associated with COVID-19 outcomes, such as COVID-19 vaccination, we purposefully chose to model our analyses parsimoniously with limited covariates, as these clinical factors themselves are strongly associated with specific SDoH factors too. For example, COVID-19 vaccination, including the number of vaccinations, is strongly associated with access to healthcare [27]. Additionally, other clinical factors, such as obesity, are highly correlated with, and act as possible upstream causes of, clinical comorbidities [28]. Given the pervasive role SDoH factors may play for various clinical contexts, and until more sophisticated modeling approaches are developed, such as those that allow moderation or mediation effects of various clinical factors, we chose to minimize the number of covariates included in our analyses. We detail these and other descriptive variables available in N3C in Supplementary Text.

### Statistical analyses

We use descriptive statistics, employing counts and proportions for categorical variables and median with interquartile range (IQR) for continuous variables, for the groups stratified by HIV status and race/ethnicity. Our overall analytic approach was to build stepwise, hierarchically nested additive models, that layered on additional modeling complexity and helped us address our three additive research questions. First, we ascertained whether an adverse association existed between each SDoH factor individually and COVID-19-related hospitalization and if that association persisted as we added additional covariates hierarchically to the model. Second, we analyzed the independent effects of each SDoH factor by HIV status; these models we call our “HIV-stratified” models, and they explicitly exclude race/ethnicity so that we can examine any race/ethnicity relationships more thoroughly in our third step. Third, we built a final model that comprised all three SDoH factors and HIV status, to assess their joint additive effects for COVID-19-related hospitalization, stratified by race/ethnicity (termed as “race/ethnicity-stratified” models). We stress presenting unadjusted, alongside adjusted, estimates as the true influence of SDoH factors for COVID-19 outcomes may indeed be pervasive; as noted earlier, clinical factors themselves may be closely associated with SDoH, and, thus, when adjusting for clinical comorbidity burden, which may be intermediate factors on the causal pathways, some bias may be introduced in our models.

Each of the three steps in our overall analytic approach accounts for heterogeneity by data partners. The N3C dataset comprises diverse clinical settings within each healthcare system, resulting in substantial heterogeneity. To handle this variability, we employed generalized linear mixed-effects models (GLMMs) tailored to accommodate and estimate associations within each healthcare system, by including random (referent log-odds) intercepts, rather than providing population-wide averages across systems [29]. Analyses were conducted using Apache Spark, SQL, Python (v3.7.12), R (v3.6.3), along with select R packages: exactci (v1.3); geepack (v1.3), glmnet (v4.1), lme4 (v1.1); metafor (v.2.4), tidyverse (v1.3.1) in the N3C Enclave (Palantir Foundry) environment. We provide specifics of our three step approach, including implementation details of model-fitting, in Supplementary Text.

## Results

### Individual-level SDoH reporting distributions within the entire N3C

Among all the individuals with at least one individual-level SDoH assessment in their record in the entire N3C system (1.5M), the source population for this analysis, the proportion of contributing data partners (*n* = 28) reporting on each SDoH domain was as follows: most reported on access to healthcare (e.g., 24 (86%) on transportation), economic instability (e.g., 25 (89%) on food insecurity, 21 (75%) on financial strain, and 7 (25%) on housing), and social cohesion (e.g., 19 (68%) on social connectedness). Across all individual-level SDoH categories, data partners reported a median of 8% (IQR 1, 15%) of their included individuals in N3C, with only one partner reporting for at least one SDoH assessment for 87% of their included individuals in N3C. Thus, the reporting on individual-level SDoH assessments was overall sparse in N3C and heterogeneous among data partners.

### Analytic sample characteristics

**Figure 1.**
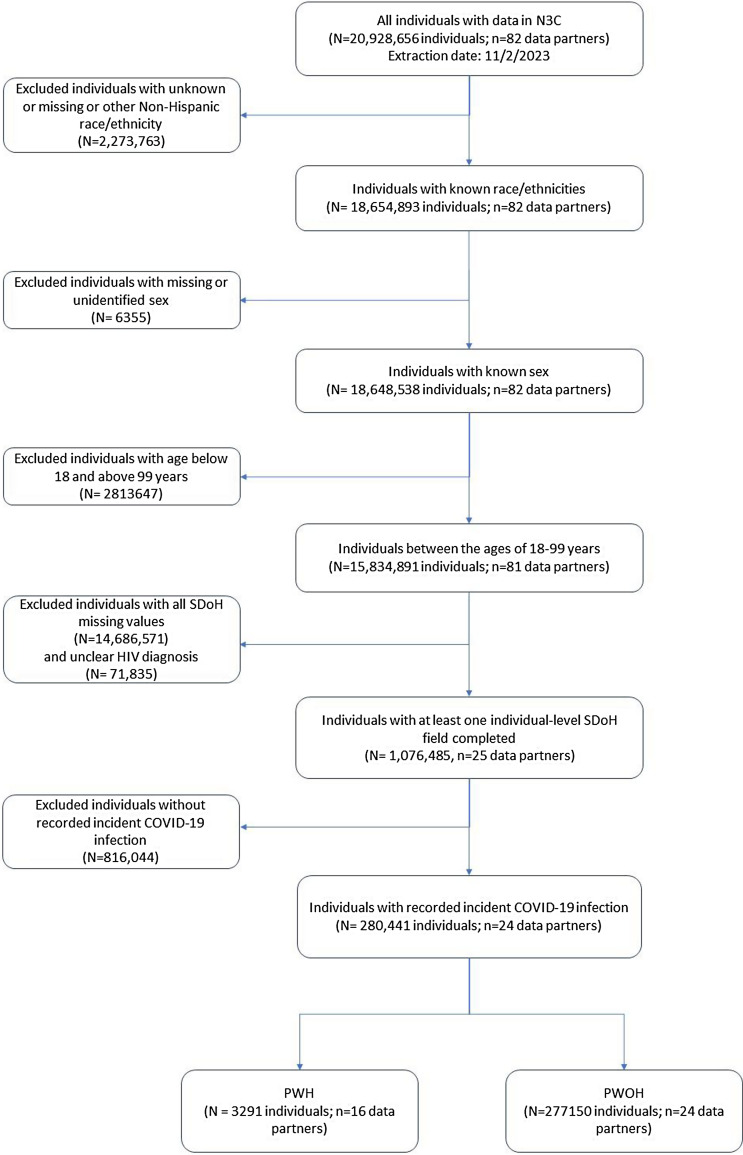
Study inclusion flowchart for analytic sample from U.S. N3C, January 2020–November 2023. Abbreviations: N3C = National COVID Cohort Collaborative; SDoH = social determinants of health; PWH = people with HIV; PWoH = people without HIV; HIV = human immunodeficiency virus.

Of the 20.9 million patients in N3C, 15.8 million were between 18 and 99 years old and had race/ethnicity and sex data available (Fig. 1). Of those, 1.1 million (6.80%) had at least one individual-level SDoH assessment in their record, of which 280,441 (26.05%) had incident COVID-19 infection. Thus, our analytic sample included 280,441 individuals from 24 data partner sites, where 3,291 (1.17%) were PWH and 277,150 (98.83%) were PWoH. The percentage of racialized minority individuals was higher among PWH vs. PWoH (e.g., 760 (23.09%) vs. 37,358 (13.47%), respectively, being NH-Black/African American; Table 1 and Supplementary Table 2). Overall, COVID-19-related hospitalizations occurred in 31,510 (11.23%) individuals, of which 302 (9.17%) occurred among PWH and 31,208 (11.26%) among PWoH.

**Table 1.** Baseline characteristics by race/ethnicity categories among COVID-19 positive individuals included in analysis in the U.S. National COVID-19 Cohort Collaborative (N3C), January 2020–November 2023 (*N* = 280,441)

Variable category	Category level	NH- American Indian or Alaska Native (AIAN) (*N* = 1,608)	NH-Asian American, Native Hawaiian, or Pacific Islander (AANHPI) (*N* = 6,039)	NH-Black African American (*N* = 38,118)	Hispanic/Latinx of any race (*N* = 21,990)	NH-White (*N* = 212,686)	Overall [*N* = 280,441, *n* = 24 data partner sites]
HIV positive status	Positive	<20 (*)	96 (1.59)	761 (2.00)	213 (0.97)	2212 (1.04)	3291 (1.17)
Sex	Male	547 (34.02)	2167 (35.88)	12,329 (32.34)	7768 (35.32)	84,993 (39.96)	107,804 (38.44)
	Female	1061 (65.98)	3872 (64.12)	25,789 (67.65)	14,222 (64.67)	127,693 (60.04)	172,637 (61.55)
Age (years)	18-44	561 (34.89)	2855 (47.28)	13,337 (34.99)	10,224 (46.50)	58,784 (27.64)	85,761 (30.58)
	45-64	561 (34.89)	1802 (29.84)	12,694 (33.30)	6302 (28.65)	61,584 (28.95)	111,737 (39.84)
	>65	486 (30.22)	1382 (22.88)	12,087 (31.71)	5464 (24.85)	92,318 (43.40)	82,943 (29.57)
CCI score^0^, median (IQR)		2 (0,5)	1 (0,2)	2 (0,5)	1 (0,3)	1 (0,4)	1 (0,4)
Insurance coverage by zip code for individuals aged 19-64^1^	High (>93.1)	330 (20.52)	2294 (37.99)	4134 (10.84)	3790 (17.23)	79,851 (37.54)	90,399 (32.23)
	Medium (<93.1, >86.0)	406 (25.25)	2005 (33.20)	12,644 (33.17)	8757 (39.82)	73,347 (34.49)	97,159 (34.64)
	Low (<86.0)	716 (44.53)	822 (13.61)	19,191 (50.34)	7436 (33.81)	44,433 (20.89)	72,598 (25.89)
	Missing	156 (9.70)	918 (15.20)	2149 (5.64)	2007 (9.13)	15,055 (7.08)	20,285 (7.23)
Region of participant resident	Northeast	25 (1.55)	511 (8.46)	2976 (7.80)	7458 (33.91)	7141 (3.36)	18,111 (6.46)
	Midwest	633 (39.36)	2538 (42.03)	8730 (22.90)	5138 (23.36)	108,091 (50.82)	125,130 (44.62)
	South	739 (45.96)	2289 (37.90)	23,928 (62.77)	4716 (21.45)	73,631 (34.62)	105,303 (37.55)
	West	119 (7.40)	361 (5.98)	1071 (2.81)	2823 (12.84)	15,924 (7.49)	20,298 (7.24)
	Missing	92 (5.72)	340 (5.63)	1413 (3.71)	1855 (8.43)	7899 (3.71)	11,599 (4.13)
BMI^2^	Underweight	<20 (*)	37 (0.61)	174 (0.46)	101 (0.46)	762 (0.36)	1078 (0.38)
	Healthy weight	166 (10.32)	1270 (21.03)	2948 (7.73)	2123 (8.69)	25,574 (12.02)	32,090 (11.44)
	Overweight	371 (23.07)	2071 (34.29)	6655 (17.46)	5135 (23.35)	55,381 (26.04)	69,613 (24.82)
	Obese	1003 (62.37)	1824 (30.20)	25,932 (68.03)	12,670 (57.62)	121516 (57.13)	162,945 (58.10)
	Missing	64 (3.98)	837 (13.86)	2404 (6.31)	1957 (8.90)	9453 (4.44)	14,715 (5.25)
Number of COVID-19 vaccinations	0	876 (54.48)	2277 (37.70)	17,732 (46.52)	13,766 (62.60)	99,395 (46.73)	134,046 (47.80)
	1	111 (6.90)	529 (8.76)	2586 (6.78)	1430 (6.50)	17,961 (8.44)	22,617 (8.06)
	2	287 (17.85)	1075 (17.80)	6254 (16.41)	3234 (14.71)	34,072 (16.02)	44,922 (16.02)
	3 or more	334 (20.77)	2158 (35.73)	11,546 (30.29)	3560 (16.19)	61,258 (28.80)	78,856 (28.12)
Access to healthcare issue	Yes	70 (4.35)	97 (1.60)	1516 (3.98)	650 (2.95)	3119 (1.47)	5452 (1.94)
	No	1285 (79.91)	4551 (75.36)	25,730 (67.50)	12,440 (56.57)	164,763 (77.45)	208743 (74.43)
	Missing	253 (15.73)	1391 (23.03)	10,872 (28.52)	8900 (40.47)	44,830 (21.08)	66,246 (23.62)
Social cohesion issue	Yes	725 (45.09)	2476 (41.02)	12,213 (32.04)	6316 (28.72)	87,602 (41.19)	109,332 (38.98)
	No	273 (16.98)	1727 (28.60)	8202 (21.51)	7447 (33.86)	66,766 (31.39)	84,415 (30.10)
	Missing	610 (37.93)	1836 (30.40)	17,703 (46.44)	8227 (37.41)	58,318 (27.42)	86,694 (30.91)
Economic stability issue	Yes	227 (14.12)	602 (9.97)	5703 (14.96)	2914 (13.25)	19,307 (9.08)	28,753 (10.25)
	No	1192 (74.13)	4600 (76.17)	26,548 (69.65)	13,554 (61.64)	169,981 (79.92)	215,875 (76.98)
	Missing	189 (11.75)	837 (13.86)	5867 (15.39)	5522 (25.11)	23,398 (11.00)	35,813 (12.78)
COVID-19 related hospitalization	Yes	209 (13.00)	414 (6.85)	7575 (19.87)	3796 (17.26)	19,516 (9.18)	31,510 (11.23)

Abbreviations: NH = Non-Hispanic/Latinx, CCI = Charlson Comorbidity Index.

*This cell count is associated with a nonzero count, that is <20. Thus, to align with N3C agreements we do not populate the corresponding proportion (%) of the value and we obfuscate** the remaining cell counts in the row to prevent any meaningful back-calculation of this nonzero but <20 cell count.

**For more detail on the obfuscation method employed with contingent cell counts, see supplement.

0 CCI: derived from binary flags for comorbidities before the first incident COVID-19 infection, excluding human immunodeficiency virus.

1 Insurance: Insurance coverage data for individuals aged 19–64, reflecting “high,” “medium,” and “low” categories, was sourced from the American Community Survey. These classifications, based on nationwide tertile cutoffs, denote varying rates of health insurance coverage within the zip code.

2 BMI categories are: underweight (<18.5), healthy weight (≥18.5 and <25), overweight (25–30), and obese (≥30).

### Individual-level SDoH reporting distributions among PWH and PWoH by race/ethnicity and sex

**Figure 2.**
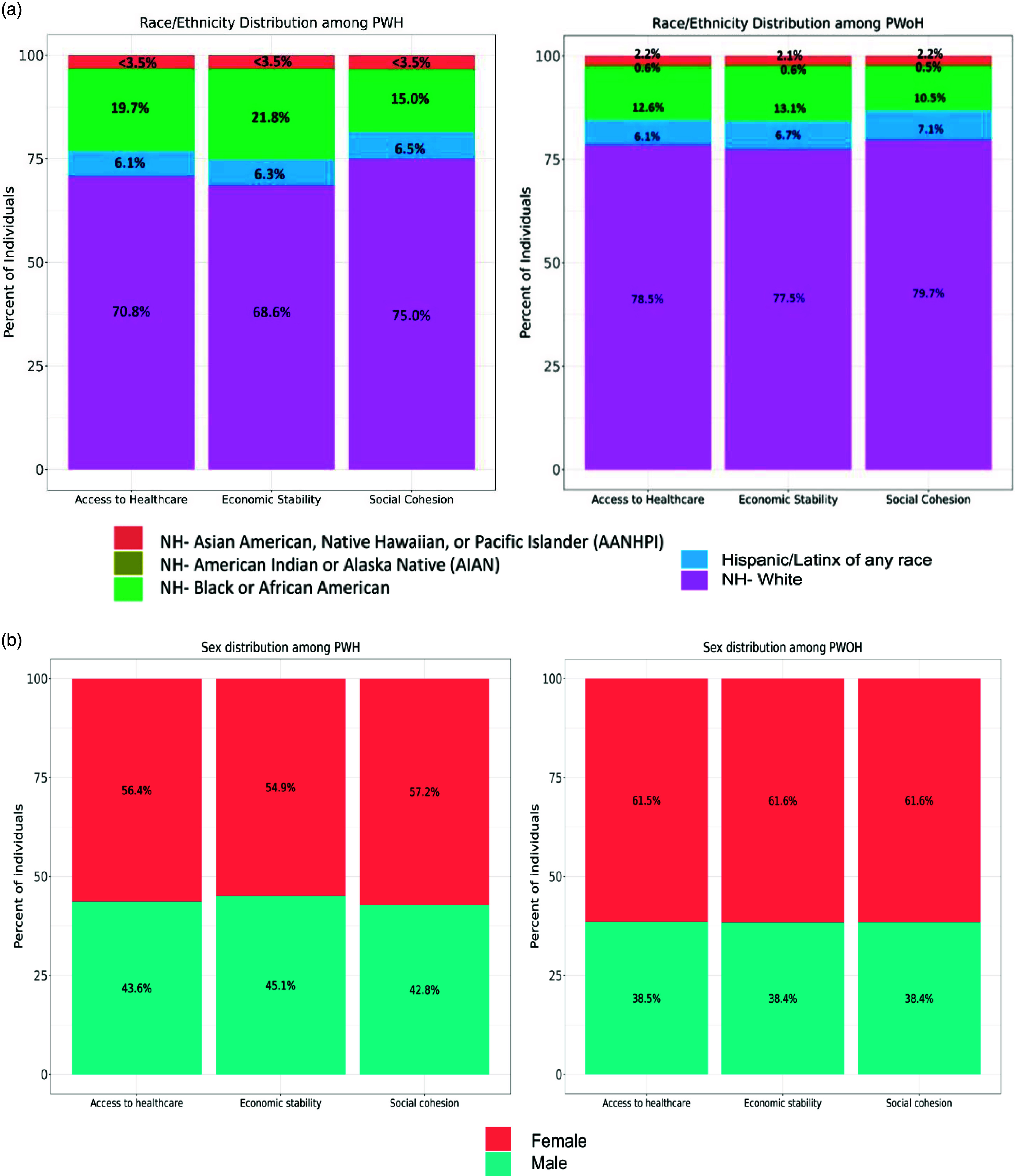
**(a)** Distribution of individual-level SDoH data reported by race/ethnicity among people with human immunodeficiency virus (HIV) (PWH) and people without HIV (PWoH) from the U.S. National COVID-19 Cohort Collaborative (N3C), January 2020–November 2023. **(b)** Distribution of individual-level social determinants of health data reported by sex among PWH and PWoH from the U.S. N3C, January 2020–November 2023. Percentage of Non-Hispanic-American Indian or Alaska Native among PWH is not reported here since cell count is associated with a nonzero count, that is <20. Thus, to align with N3C agreements, we do not populate the corresponding proportion (%) of the value.

Figure 2a summarizes SDoH factors ascertained for PWH and PWoH among different race/ethnicity groups. Across each SDoH domain, PWH had higher proportions of racialized minority individuals than PWoH. NH-Black/African American PWH exhibited higher issues with access to healthcare (19.7%), economic stability (21.8%), and social cohesion (15%) compared to their counterparts among other PWH and PWoH (e.g., 12.6% access to health services, 13.1% economic stability, and 10.5% social cohesion among NH-Black/African American PWoH).

Figure 2b summarizes SDoH factors ascertained individually for PWH and PWoH among males and females. Females consistently demonstrated higher proportions for issues with access to healthcare, economic stability, and social cohesion compared to males, and these proportions were similar among PWH and PWoH.

### Summary of step 1, hierarchically nested SDoH models for all individuals

In our first step of the modeling approach, all three SDoH domains consistently demonstrated statistically significant associations with COVID-19-related hospitalizations, even after successive, hierarchically nested adjustments (Table 2). For access to healthcare issues, the univariate model revealed a significant association with an odds ratio (OR) of 1.97 (95% confidence interval [CI]: 1.83, 2.11). When adjusted for age, sex, CCI, and HIV, the adjusted OR was 1.85 (1.72, 1.20), and incrementally adjusting for race/ethnicity yielded an OR of 1.71 (1.59, 1.85). Similarly, economic instability (univariate OR [uOR] 1.43 (1.38, 1.50), adjusted for age, sex, CCI, and HIV OR 1.48 (1.42, 1.54), further adjusted for race/ethnicity OR 1.36 (1.31, 1.42) and social cohesion (uOR 1.52 (1.47, 1.59), adjusted for age, sex, CCI, and HIV OR 1.41 (1.36, 1.47), further adjusted for race/ethnicity OR 1.39 (1.34, 1.45) exhibited notable associations with hospitalizations.

**Table 2.** Results of hierarchically nested models for each individual-level social determinants of health (SDoH) factor and COVID-19-related hospitalization for all individuals with incident COVID-19 infection in the U.S. National COVID-19 Cohort Collaborative (N3C), January 2020–November 2023 (*N* = 280,441)

Category	Model 1: Univariate OR (95% CI)	Model 2: M1 + age, sex, CCI OR (95% CI)	Model 3: M2 + HIV OR (95% CI)	Model 4: M3 + race/ethnicity OR (95% CI)
Access to healthcare issue	**1.97 (1.83, 2.11)**	**1.85 (1.72, 2.00)**	**1.85 (1.72, 1.20)**	**1.71 (1.59, 1.85)**
Economic stability issue	**1.43 (1.38, 1.50)**	**1.48 (1.42, 1.54)**	**1.48 (1.42, 1.54)**	**1.36 (1.31, 1.42)**
Social cohesion issue	**1.52 (1.47, 1.59)**	**1.41 (1.36, 1.47)**	**1.41 (1.36, 1.47)**	**1.39 (1.34, 1.45)**

Abbreviations: M1 = Model 1; M2 = Model2; M3 = Model 3; CCI = Charlson Comorbidity Index; OR = odds ratio; CI = confidence interval.

*Note*: Models are sequentially adjusted for covariates.

M1: Univariate or “unadjusted” odds ratios obtained via modeling each SDoH factor (as exposure) and COVID-19-related hospitalization (as outcome) using mixed-effects logistic regression or generalized linear mixed-effects models (GLMMs), with random effects restricted to a random intercept, i.e., referent log-odds, for each unique data partner.

M2: Adjusts M1 for age (categorical), sex, and Charlson Comorbidity Index (CCI, continuous).

M3: Further adjusts M2 by including HIV status.

M4: Further adjusts M3 by including race/ethnicity.

**Bold text** indicates estimates with *p*-values < 0.05.

### Summary of step 2, HIV-stratified analyses

In our second step of the analyses stratified by HIV status, significant associations were found between individual-level SDoH factors and COVID-19-related hospitalizations (Table 3). Among PWH, economic stability was significantly associated with hospitalizations (uOR 1.35 (95% CI: 1.01, 1.82), adjusted OR [aOR] 1.41 (95% CI:1.37, 1.49)). Among PWoH, access to healthcare (uOR 1.98 (1.85, 2.13), aOR 1.87 (1.73, 2.02)), economic instability (uOR 1.43 (1.37, 1.49), aOR 1.48 (1.42, 1.52)), and social connectedness (uOR 1.53 (1.47, 1.60), aOR 1.42 (1.36, 1.47)) were significantly associated with hospitalizations. Overall, these results suggest that the impact of SDoH factors on COVID-19-related hospitalizations varied between PWH and PWoH, with larger effect sizes generally observed in PWoH and only economic stability issues emerging as statistically significant among PWH. Covariate estimates are found in Supplementary Table 3.

**Table 3.** Results of modeling each individual-level social determinants of health (SDoH) factor and COVID-19-related hospitalization stratified by human immunodeficiency virus (HIV) status in the U.S. National COVID-19 Cohort Collaborative (N3C), January 2020–November 2023 (N = 280,441)

Category	PWH (n = 3291)		PWoH (n = 277,150)	
	Unadjusted OR^1^ (95% CI)	Adjusted OR^2^ (95% CI)	Unadjusted OR^1^ (95% CI)	Adjusted OR^2^ (95% CI)
Access to healthcare issue	1.03 (0.60, 1.74)	0.99 (0.56, 1.73)	**1.98 (1.85, 2.13)**	**1.87 (1.73, 2.02)**
Economic stability issue	**1.35 (1.01, 1.82)**	**1.41 (1.03, 1.92)**	**1.43 (1.37, 1.49)**	**1.48 (1.42, 1.54)**
Social cohesion issue	1.04 (0.74, 1.46)	0.99 (0.70, 1.40)	**1.53 (1.47, 1.60)**	**1.42 (1.36, 1.47)**

Abbreviations: PWH = people with HIV; PWoH = people without HIV; OR = odds ratio; CI = confidence interval.

*Note*: **Bold text** indicates estimates with *p*-values<0.05.

1 Unadjusted odds ratios obtained via modeling each SDoH factor (as exposure) and COVID-19-related hospitalization (as outcome) using mixed-effects logistic regression or generalized linear mixed-effects models, with random effects restricted to a random intercept, i.e., referent log-odds, for each unique data partner.

2 Adjusted odds ratios obtained via modeling each SDoH factor (as exposure) and COVID-19-related hospitalization (as outcome) using mixed-effects logistic regression, with random effects restricted to a random intercept, i.e., referent log-odds, for each unique data partner, and adding regression terms for age (categorical), sex, and Charlson Comorbidity Index (CCI, continuous).

### Summary of step 3, race/ethnicity-stratified analyses

**Figure 3.**
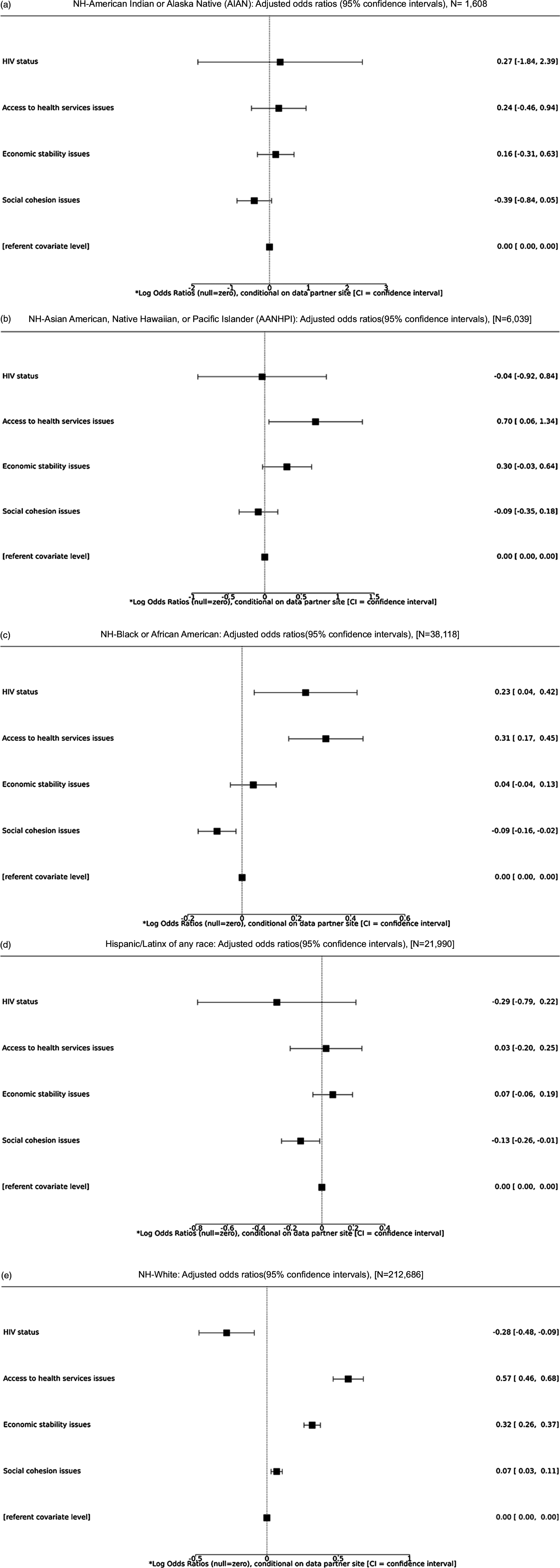
Forest plots of adjusted odds ratios from modeling of individual-level social determinants of health factors, human immunodeficiency virus (HIV) status, and COVID-19-related hospitalization stratified by race/ethnicity in the U.S. National COVID-19 Cohort Collaborative (N3C), January 2020–November 2023. (A) Non-Hispanic-American Indian or Alaska Native: Adjusted odds ratios (95% confidence intervals), *N* = 1,608. (B) NH-Asian American, Native Hawaiian, or Pacific Islander: Adjusted odds ratios (95% confidence intervals), [*N* = 6,039]. (C) NH-Black or African American: Adjusted odds ratios (95% confidence intervals), [*N* = 38,118]. (D) Hispanic/Latinx of any race: Adjusted odds ratios (95% confidence intervals), [*N* = 21,990]. (E) NH-White: Adjusted odds ratios (95% confidence intervals), [*N* = 212,686]. Generated with adjusted mixed-effects logistic regression or generalized linear mixed-effects models, with random effects restricted to a random intercept, i.e., referent log-odds, for each unique data partner. We thus accounted for data partner sites along with covariates (age, sex, Charlson Comorbidity Index among others reported across Table 3 and Supplementary Table 3); model implementations use package lme4 v.1.1 while plotting employs package metafor v.2.4 using R v.3.6.3 within the Palantir Foundry hosted N3C Enclave.

In our third step of analyzing the associations between SDoH and COVID-19-related hospitalizations across various racial/ethnic groups when accounting for HIV status, distinct patterns emerged (Fig. 3, Table 4). Below we highlight statistically significant findings.

**Table 4.** Results of modeling jointly individual-level social determinants of health (SDoH) factors, human immunodeficiency virus (HIV) status, and COVID-19-related hospitalization stratified by race/ethnicity in the U.S. National COVID-19 Cohort Collaborative (N3C), January 2020–November 2023 (N = 280,441)

	NH-American Indian or Alaska Native (*N* = 1,608)		NH-AANHPI (*N* = 6,039)		NH-Black/African American (*N* = 38,118)		Hispanic/Latinx of any race (*N* = 21,990)		NH-White (*N* = 212,686)	
	Unadjusted OR^1^ (95% CI)	Adjusted OR^2^ (95% CI)	Unadjusted OR^1^ (95% CI)	Adjusted OR^2^ (95% CI)	Unadjusted OR^1^ (95% CI)	Adjusted OR^2^ (95% CI)	Unadjusted OR^1^ (95% CI)	Adjusted OR^2^ (95% CI)	Unadjusted OR^1^ (95% CI)	Adjusted OR^2^ (95% CI)
HIV positive	0.97 (0.12, 7.90)	1.31 (0.16, 10.9)	1.07 (0.46, 2.49)	0.96 (0.40, 2.32)	**1.43 (1.20, 1.71)**	**1.26 (1.04, 1.53)**	0.88 (0.54, 1.44)	0.75 (0.45, 1.24)	**0.76 (0.64, 0.90)**	**0.75 (0.62, 0.91)**
Access to healthcare issues	1.35 (0.68, 2.68)	1.27 (0.63, 2.56)	**2.00 (1.08, 3.70)**	**2.00 (1.06, 3.80)**	**1.43 (1.26, 1.63)**	**1.36 (1.19, 1.56)**	1.04 (0.84, 1.30)	1.03 (0.82, 1.29)	**1.90 (1.72, 2.10)**	**1.77 (1.59, 1.96)**
Economic instability	1.12 (0.72, 1.76)	1.17 (0.73, 1.88)	**1.67 (1.21, 2.29)**	1.35 (0.97, 1.90)	1.02 (0.95, 1.11)	1.04 (0.96, 1.13)	1.02 (0.90, 1.15)	1.07 (0.94, 1.21)	**1.29 (1.22, 1.36)**	**1.37 (1.30, 1.45)**
Social cohesion issue	0.76 (0.50, 1.13)	0.68 (0.43, 1.10)	0.92 (0.72, 1.21)	0.91 (0.70, 1.19)	0.94 (0.88, 1.01)	**0.91 (0.85, 0.97)**	0.92 (0.82, 1.04)	**0.87 (0.77, 0.99)**	**1.10 (1.06, 1.14)**	**1.07 (1.03, 1.11)**

Abbreviations: NH = Non-Hispanic/Latinx; AANHPI = Asian American, Native Hawaiian, or Pacific Islander.

1 “Unadjusted” here means no age/sex/ Charlson Comorbidity Index terms included regardless**.

2 “Adjusted” here means *additionally* adjusted for age/sex/ Charlson Comorbidity Index.

Primary Multivariable analysis results by racial/ethnic groups: Data Partner Site Conditional* Odds Ratios (and 95% confidence intervals) estimated via generalized linear mixed-effects model-fitted models (random intercepts by data partner site); mean model features selected per generalized linear model-cross-validation-specified regularization**.

* “Conditional” in the sense that all odds ratio estimates are interpreted as *conditional on* the referent log-odds of hospitalization predicted for each data partner healthcare system, as modeling approach to accommodate heterogeneity across systems.

**Mean models selected via cross-validation per LASSO (L1 regularized) fits at minimum (optimal) lambda value… then maintaining age-/sex-/ Charlson Comorbidity Index-adjustment variables for *further* “adjusted” estimates; “_” = covariate not selected.

Access to healthcare issues showed significant association with hospitalizations among various racialized groups. NH-AANHPI (uOR 2.00 (95% CI: 1.08, 3.70), aOR 2.00 (CI 1.06, 3.80)) and NH-White (uOR 1.90 (1.72, 2.10), aOR 1.77 (1.59, 1.96)) groups exhibited the highest impact. NH-Black/African American (uOR 1.43 (1.26, 1.19), aOR 1.36 (1.19, 1.56)) group had lower impact.

Economic instability showed significant associations with certain racialized groups, notably among NH-AANHPI (uOR 1.67 (1.21, 2.29), aOR 1.35 (0.97, 1.90)) and NH-White (uOR 1.29 (1.22, 1.36), aOR 1.37 (1.30, 1.45)) groups.

Social cohesion issues showed significant associations only among NH-White (uOR 1.10 (1.06, 1.14), aOR 1.07 (1.03, 1.11)). Among NH-Black/African American (uOR 0.94 (0.88, 1.01), aOR 0.91 (0.85, 0.97)) and Hispanic/Latinx (uOR 0.92 (0.82, 1.04), aOR 0.87 (0.77, 0.99)) groups, lower odds existed for hospitalizations.

In these fully adjusted models, living with HIV was only significantly associated with hospitalizations among NH-Black/African American groups (uOR 1.43 (1.20, 1.71), aOR 1.26 (1.04, 1.53)). In contrast, living with HIV was protective among NH-White group (uOR 0.76 (0.64, 0.90), aOR 0.75 (0.62, 0.91)). Covariate estimates are found in Supplementary Table 4, and intermediate model estimates in Supplementary Table 5.

## Discussion

Our study reveals significant insights into the influence of individual-level SDoH factors on COVID-19-related hospitalizations for both PWH and PWoH. In our initial modeling, key SDoH variables, such as access to healthcare, economic instability, and social cohesion, uniformly emerged as persistent factors associated with higher odds of hospitalization across both cohorts. This underscores the pervasive impact of these factors irrespective of HIV status or race/ethnicity groups. Our multivariable analysis showed that living with HIV increases the likelihood of COVID-19 hospitalization among NH-Black/African Americans, highlighting their heightened vulnerability, even when factoring in SDoH. Our analysis, therefore, not only reinforces the importance of addressing SDoH in public health policies but also calls for a heightened focus on the specific needs of PWH during pandemic responses. Our approach of using individual-level SDoH data, departing from conventional area-level analyses, enhances the granularity of our understanding of big data and elevates the precision with which targeted interventions can be implemented for specific individuals–this, arguably, is a novel use of big data for precision public health.

In regards to living with HIV, our analyses revealed some interesting findings. First, SDoH issues were more prevalent among PWH than PWoH. It is well established that people at risk of HIV face significant social vulnerabilities (e.g., homelessness), and living with HIV can engender additional vulnerabilities (e.g., strained social networks) [30,31]. Second, we observed that the overall proportions of COVID-19-related hospitalizations were lower among PWH than PWoH (9.17% vs. 11.26%, respectively). This is intriguing for several reasons and suggests that our analytic sample might be biased in various ways, as our prior work has shown higher risk of adverse COVID-19 outcomes, including hospitalizations, among PWH [24]. It is possible that PWH, who also have individual-level SDoH recorded in EHR, are more engaged in care than PWoH and subsequently get their social vulnerability addressed [32]. While some HIV medications, such as protease inhibitors, have been hypothesized to help treat COVID-19, and, therefore, prevent COVID-19-related hospitalizations [33], we do not anticipate sufficiently large exposures to such medications to account for these group effects. Third, in the HIV-stratified models, economic instability was the most impactful SDoH factor among PWH. Among PWH, there is increased vulnerability to workplace discrimination, compromised job security, and heightened barriers to employment opportunities potentially leading to economic instability issues [34]. Fourth, and most profound arguably, is that in our multivariable, race/ethnicity-stratified models, living with HIV, despite accounting for all the SDoH factors, was associated with hospitalizations only among NH-Black/African American adults, and, in fact, appeared protective among NH-White adults. This likely signals the profound disparities in living with HIV among the NH-Black/African American communities; NH-Black/African Americans account for 40% of new infections, compared to 25% among NH-Whites [9]. Admittedly, that living with HIV was protective among NH-Whites not only highlights a stark contrast but feels like an affront to health equity work; potentially NH-White PWH might be experiencing higher engagement and receiving better quality care than racialized minority PWH [35]. This situation underscores ongoing racial inequities in healthcare; despite progress over three decades, there’s much to do in enhancing care for racialized minority PWH.

We found that poor access to care and economic instability independently contributed to higher odds of COVID-19-related hospitalizations, even when accounting for living with HIV and other covariates, which carries profound implications for public health policy and practice. While poor access to care was significant for NH-AANHPI, NH-Black/African American, and NH-White populations, it was non-significant for the NH-AIAN and Hispanic/Latinx of any race groups. These latter groups are known to have significant access to care issues. However, numerous intersecting factors, which we may have not captured well, affect access to care for these groups including high rates of rurality, low health literacy, and healthcare policy [36,37]. Further, significant heterogeneity exists in Latinx ethnicity groups and among NH-AIAN subpopulations (i.e., by tribe and regionality) and, as such, our inability to explore subgroup associations may have masked existing inequities within these groups [38]. Poor access to care is not just a health issue, but a reflection of broader systemic inequities that can exacerbate the severity of disease outcomes [39]. Similarly, economic instability, often a result of and contributing to health disparities, creates a cascade of challenges that hinder individuals’ ability to seek timely medical attention and adhere to COVID-19 prevention measures [40]. The study’s focus on three SDoH domains (access to healthcare, economic stability, and social cohesion) likely overlooks other critical factors, such as within other domains of access to and quality of education and neighborhood and built environment characteristics, which highlights the complexity of and the need to further research to explore a broader range of social vulnerability. Nonetheless, the independence of these factors from HIV status shows that societal factors broadly impact health outcomes, highlighting the need for public health strategies that address both healthcare and social inequalities.

Our analysis of COVID-19-related hospitalizations among racial/ethnic minorities unearthed some unintuitive findings. Lack of social support is known to have an adverse impact on health outcomes [41]. Some studies have demonstrated that strong social support networks can significantly improve health, often helping to narrow racial disparities in health outcomes [42]. However, in the context of COVID-19, a lack of social support appears to be acting as a protective factor against hospitalization for some racialized minorities in our analysis. This paradoxical finding demands a closer examination of how social support is conceptualized and measured. In this specific study, social support was gauged through indicators such as marital status and membership in social organizations. This approach may not fully capture the essence of social support within diverse minority communities. It has long been established that defining and measuring social support is challenging [43]. There are several possible explanations for this phenomenon. First, the traditional measures of social support might not adequately reflect the support systems within minority communities, which may instead rely more on informal networks. Second, in the context of a highly infectious disease like COVID-19, traditional forms of social support involving close physical contact or group gatherings could inadvertently increase exposure risk, leading to higher hospitalization rates. Moreover, the cultural context of social support can vary significantly across different ethnic groups. For example, in some cultures, social support is not just about having a large social network, but also about the quality and nature of support provided [44].

The use of individual-level variables in our study represents a novel approach to health disparities research. In contrast to studies based on area-level SDoH, which, for instance, can fail to capture about 42% of people living in deprived conditions within otherwise privileged areas [45], our individual-level analysis has the potential to capture a greater proportion of those with SDoH needs independent of their area-level data. This methodology allowed us to capture a nuanced picture of each individual’s social drivers, shedding light on how these factors interplay with health outcomes. There is growing interest in the use of big data and analytics to support targeted public health interventions–named precision public health [46]. This was particularly evident during COVID-19 in the U.S. [46]. While precision public health has its supporters and negators [47], the enthusiasm to leverage data from EHR for public health, not just clinical care, is growing [48]. Our work using individual-level SDoH captured in a large EHR repository demonstrates the power of using such big data to help develop targeted interventions for specific populations, such as PWH.

The study, while comprehensive in many aspects, has limitations. A primary constraint in our study within the N3C domain is the limited overlap between the full cohort of COVID-19 individuals and those with recorded responses to the specific set of SDoH questions harmonized for our analysis. This gap raises concerns about the representativeness of the study cohort, potentially impacting the generalizability of our findings. Furthermore, the study is inherently limited to individuals who utilize healthcare services. Hence, those who are likely most vulnerable to adverse SDoH are not fully captured in EHR cohorts, thus overlooking these relationships for those most vulnerable. A second limitation regards the anchoring of our primary outcome, COVID-19-related hospitalization, on a COVID-19 diagnosis documented with the EHR; with the advent of home-based COVID-19 testing, it is possible that incidence of COVID-19 is under-ascertained. We do not believe this misclassification to be differential by our exposure groups. Relatedly, despite controlling for several covariates, unmeasured factors, or residual confounding, such as specific health behaviors or the quality of healthcare received, could still bias our findings. A third limitation is that we did not account for any HIV-related factors, such as CD4 count and viral suppression, which we have shown to be associated with COVID-19 outcomes [49]. However, as already noted before, clinical measures, including these for HIV, themselves are strongly associated with SDoH factors in PWH [50], and additional modeling strategies need to be developed to better account for the complex relationships between SDoH factors, clinical variables, and outcomes. Despite its limitations, our paper is strengthened by the broad U.S. representation from the N3C repository’s multi-system data, minimizing single-site biases. Moreover, our study stands out for its individual-level analysis, encompassing several categories outlined in the Healthy People 2030 framework. This detailed analysis enhances the robustness of our findings and provides a nuanced understanding of the interplay between SDoH, HIV, and COVID-19 across diverse populations and healthcare contexts.

## Conclusion

In our study, we examined the impact of individual-level SDoH on COVID-19-related hospitalizations, with a focus on PWH and PWoH across racialized communities. Our findings reveal that key SDoH factors, such as poorer access to care, economic instability, and limited social connectedness, are significantly associated with hospitalizations for both groups, highlighting their pervasive influence. Crucially, the study also uncovers that living with HIV independently exacerbates the likelihood of COVID-19-related hospitalizations within NH-Black/African Americans, even when accounting for the impact of SDoH variables. This points to a unique vulnerability among racialized minority PWH in the context of the COVID-19 pandemic, underscoring the need for public health policies to address these specific challenges. Lastly, our innovative approach, moving away from conventional area-level analyses to a more individualized examination of SDoH impacts, offers a nuanced and granular understanding of the interplay between SDoH, living with HIV, and COVID-19 outcomes. Our study advances existing knowledge and signals a major shift in public health strategies, advocating for personalized, data-driven methods in crisis management, particularly for highly vulnerable groups, to ensure responses are customized for diverse community needs.

## Supporting information

Vaidya et al. supplementary material Vaidya et al. supplementary material

## Supplementary material

The supplementary material for this article can be found at https://doi.org/10.1017/cts.2024.550

## N3C attribution

The analyses described in this publication were conducted with data or tools accessed through the NCATS N3C Data Enclave https://covid.cd2h.org and N3C Attribution & Publication Policy v 1.2-2020-08-25b supported by NCATS U24 TR002306, Axle Informatics Subcontract: NCATS-P00438-B. Individual authors were supported by the following funding sources: NIMH R01131542 (PI Rena C. Patel). The funding sources or study sponsors had no role in study design; in the collection, analysis, and interpretation of data; in the writing of the report; and in the decision to submit the paper for publication. This research was possible because of the patients whose information is included within the data and the organizations (https://ncats.nih.gov/n3c/resources/data-contribution/data-transfer-agreement-signatories) and scientists who have contributed to the ongoing development of this community resource [https://doi.org/10.1093/jamia/ocaa196].

## Disclaimer

IRB: The N3C data transfer to NCATS is performed under a Johns Hopkins University Reliance Protocol # IRB00249128 or individual site agreements with NIH. The N3C Data Enclave is managed under the authority of the NIH; information can be found at https://ncats.nih.gov/n3c/resources.

## Data partners with released data

The following institutions whose data is released or pending:

Available: Advocate Health Care Network — UL1TR002389: The Institute for Translational Medicine (ITM) • Aurora Health Care Inc. — UL1TR002373: Wisconsin Network For Health Research • Boston University Medical Campus — UL1TR001430: Boston University Clinical and Translational Science Institute • Brown University — U54GM115677: Advance Clinical Translational Research (Advance-CTR) • Carilion Clinic — UL1TR003015: iTHRIV Integrated Translational health Research Institute of Virginia • Case Western Reserve University — UL1TR002548: The Clinical & Translational Science Collaborative of Cleveland • Charleston Area Medical Center — U54GM104942: West Virginia Clinical and Translational Science Institute (WVCTSI) • Children’s Hospital Colorado — UL1TR002535: Colorado Clinical and Translational Sciences Institute • Columbia University Irving Medical Center — UL1TR001873: Irving Institute for Clinical and Translational Research • Dartmouth College — None (Voluntary) Duke University — UL1TR002553: Duke Clinical and Translational Science Institute • George Washington Children’s Research Institute — UL1TR001876: Clinical and Translational Science Institute at Children’s National (CTSA-CN) • George Washington University — UL1TR001876: Clinical and Translational Science Institute at Children’s National (CTSA-CN) • Harvard Medical School — UL1TR002541: Harvard Catalyst • Indiana University School of Medicine — UL1TR002529: Indiana Clinical and Translational Science Institute • Johns Hopkins University — UL1TR003098: Johns Hopkins Institute for Clinical and Translational Research • Louisiana Public Health Institute — None (Voluntary) • Loyola Medicine — Loyola University Medical Center • Loyola University Medical Center — UL1TR002389: The Institute for Translational Medicine (ITM) • Maine Medical Center — U54GM115516: Northern New England Clinical & Translational Research (NNE-CTR) Network • Mary Hitchcock Memorial Hospital & Dartmouth Hitchcock Clinic — None (Voluntary) • Massachusetts General Brigham — UL1TR002541: Harvard Catalyst • Mayo Clinic Rochester — UL1TR002377: Mayo Clinic Center for Clinical and Translational Science (CCaTS) • Medical University of South Carolina — UL1TR001450: South Carolina Clinical & Translational Research Institute • MITRE Corporation — None (Voluntary) • Montefiore Medical Center — UL1TR002556: Institute for Clinical and Translational Research at Einstein and Montefiore • Nemours — U54GM104941: Delaware CTR ACCEL Program • NorthShore University HealthSystem — UL1TR002389: The Institute for Translational Medicine (ITM) • Northwestern University at Chicago — UL1TR001422: Northwestern University Clinical and Translational Science Institute • OCHIN — INV-018455: Bill and Melinda Gates Foundation grant to Sage Bionetworks • Oregon Health & Science University — UL1TR002369: Oregon Clinical and Translational Research Institute • Penn State Health Milton S. Hershey Medical Center — UL1TR002014: Penn State Clinical and Translational Science Institute • Rush University Medical Center — UL1TR002389: The Institute for Translational Medicine (ITM) • Rutgers, The State University of New Jersey — UL1TR003017: New Jersey Alliance for Clinical and Translational Science • Stony Brook University — U24TR002306 • The Alliance at the University of Puerto Rico, Medical Sciences Campus — U54GM133807: Hispanic Alliance for Clinical and Translational Research (The Alliance) • The Ohio State University — UL1TR002733: Center for Clinical and Translational Science • The State University of New York at Buffalo — UL1TR001412: Clinical and Translational Science Institute • The University of Chicago — UL1TR002389: The Institute for Translational Medicine (ITM) • The University of Iowa — UL1TR002537: Institute for Clinical and Translational Science • The University of Miami Leonard M. Miller School of Medicine — UL1TR002736: University of Miami Clinical and Translational Science Institute • The University of Michigan at Ann Arbor — UL1TR002240: Michigan Institute for Clinical and Health Research • The University of Texas Health Science Center at Houston — UL1TR003167: Center for Clinical and Translational Sciences (CCTS) • The University of Texas Medical Branch at Galveston — UL1TR001439: The Institute for Translational Sciences • The University of Utah — UL1TR002538: Uhealth Center for Clinical and Translational Science • Tufts Medical Center — UL1TR002544: Tufts Clinical and Translational Science Institute • Tulane University — UL1TR003096: Center for Clinical and Translational Science • The Queens Medical Center — None (Voluntary) • University Medical Center New Orleans — U54GM104940: Louisiana Clinical and Translational Science (LA CaTS) Center • University of Alabama at Birmingham — UL1TR003096: Center for Clinical and Translational Science • University of Arkansas for Medical Sciences — UL1TR003107: UAMS Translational Research Institute • University of Cincinnati — UL1TR001425: Center for Clinical and Translational Science and Training • University of Colorado Denver, Anschutz Medical Campus — UL1TR002535: Colorado Clinical and Translational Sciences Institute • University of Illinois at Chicago — UL1TR002003: UIC Center for Clinical and Translational Science • University of Kansas Medical Center — UL1TR002366: Frontiers: University of Kansas Clinical and Translational Science Institute • University of Kentucky — UL1TR001998: UK Center for Clinical and Translational Science • University of Massachusetts Medical School Worcester — UL1TR001453: The UMass Center for Clinical and Translational Science • University Medical Center of Southern Nevada — None (voluntary) • University of Minnesota — UL1TR002494: Clinical and Translational Science Institute • University of Mississippi Medical Center — U54GM115428: Mississippi Center for Clinical and Translational Research • University of Nebraska Medical Center — U54GM115458: Great Plains IDeA-Clinical & Translational Research • University of North Carolina at Chapel Hill — UL1TR002489: North Carolina Translational and Clinical Science Institute • University of Oklahoma Health Sciences Center — U54GM104938: Oklahoma Clinical and Translational Science Institute • University of Pittsburgh — UL1TR001857: The Clinical and Translational Science Institute (CTSI) • University of Pennsylvania — UL1TR001878: Institute for Translational Medicine and Therapeutics • University of Rochester — UL1TR002001: UR Clinical & Translational Science Institute • University of Southern California — UL1TR001855: The Southern California Clinical and Translational Science Institute (SC CTSI) • University of Vermont — U54GM115516: Northern New England Clinical & Translational Research (NNE-CTR) Network • University of Virginia — UL1TR003015: iTHRIV Integrated Translational health Research Institute of Virginia • University of Washington — UL1TR002319: Institute of Translational Health Sciences • University of Wisconsin-Madison — UL1TR002373: UW Institute for Clinical and Translational Research • Vanderbilt University Medical Center — UL1TR002243: Vanderbilt Institute for Clinical and Translational Research • Virginia Commonwealth University — UL1TR002649: C. Kenneth and Dianne Wright Center for Clinical and Translational Research • Wake Forest University Health Sciences — UL1TR001420: Wake Forest Clinical and Translational Science Institute • Washington University in St Louis — UL1TR002345: Institute of Clinical and Translational Sciences • Weill Medical College of Cornell University — UL1TR002384: Weill Cornell Medicine Clinical and Translational Science Center • West Virginia University — U54GM104942: WVCTSI Submitted: Icahn School of Medicine at Mount Sinai — UL1TR001433: ConduITS Institute for Translational Sciences • The University of Texas Health Science Center at Tyler — UL1TR003167: CCTS • University of California, Davis — UL1TR001860: UCDavis Health Clinical and Translational Science Center • University of California, Irvine — UL1TR001414: The UC Irvine Institute for Clinical and Translational Science • University of California, Los Angeles — UL1TR001881: UCLA Clinical Translational Science Institute • University of California, San Diego — UL1TR001442: Altman Clinical and Translational Research Institute • University of California, San Francisco — UL1TR001872: UCSF Clinical and Translational Science Institute Pending: Arkansas Children’s Hospital — UL1TR003107: UAMS Translational Research Institute • Baylor College of Medicine — None (Voluntary) • Children’s Hospital of Philadelphia — UL1TR001878: Institute for Translational Medicine and Therapeutics • Cincinnati Children’s Hospital Medical Center — UL1TR001425: Center for Clinical and Translational Science and Training • Emory University — UL1TR002378: Georgia Clinical and Translational Science Alliance • HonorHealth — None (Voluntary) • Loyola University Chicago — UL1TR002389: The Institute for Translational Medicine (ITM) • Medical College of Wisconsin — UL1TR001436: Clinical and Translational Science Institute of Southeast Wisconsin • MedStar Health Research Institute — None (Voluntary) • Georgetown University — UL1TR001409: The Georgetown-Howard Universities Center for Clinical and Translational Science • MetroHealth — None (Voluntary) • Montana State University — U54GM115371: American Indian/Alaska Native CTR • NYU Langone Medical Center — UL1TR001445: Langone Health’s Clinical and Translational Science Institute • Ochsner Medical Center — U54GM104940: Louisiana Clinical and Translational Science (LA CaTS) Center • Regenstrief Institute — UL1TR002529: Indiana Clinical and Translational Science Institute • Sanford Research — None (Voluntary) • Stanford University — UL1TR003142: Spectrum: The Stanford Center for Clinical and Translational Research and Education • The Rockefeller University — UL1TR001866: Center for Clinical and Translational Science • The Scripps Research Institute — UL1TR002550: Scripps Research Translational Institute • University of Florida — UL1TR001427: UF Clinical and Translational Science Institute • University of New Mexico Health Sciences Center — UL1TR001449: University of New Mexico Clinical and Translational Science Center • University of Texas Health Science Center at San Antonio — UL1TR002645: Institute for Integration of Medicine and Science • Yale New Haven Hospital — UL1TR001863: Yale Center for Clinical Investigation

**IRB:** The N3C data transfer to NCATS is performed under a Johns Hopkins University Reliance Protocol # IRB00249128 or individual site agreements with NIH. The N3C Data Enclave is managed under the authority of the NIH; information can be found at ncats.nih.gov/n3c/resources. Enclave data are protected, and can be accessed for COVID-related research with an NIH-approved (1) IRB protocol and (2) institutional Data Use Request (DUR; DUR id: RP-CA3365). A detailed accounting of data protection and access tiers is found at https://ncats.nih.gov/n3c/resources/data-access. Enclave and data access instructions can be found at https://covid.cd2h.org/for-researchers; all code used to produce the analyses in this manuscript is available within the N3C Enclave to users with valid login credentials to support reproducibility.
