# Supplementary material for: Assessing associations between individual-level social determinants of health and COVID-19 hospitalizations: Investigating racial/ethnic disparities among people living with human immunodeficiency virus (HIV) in the U.S. National COVID Cohort Collaborative (N3C)
Source: J Clin Transl Sci. 2024 May 21;8(1):e107. doi: 10.1017/cts.2024.550 (PMC11408162; doi:10.1017/cts.2024.550)
Supplement: Vaidya et al. supplementary material [file S2059866124005508sup001.docx]

**Supplementary Text**

**Methods (cont.)**

*Study Design and Analytic Sample(cont.)*

In collaboration with HIV specialists on our team, we developed a four-tiered classification system to ascertain the HIV status of each person with HIV (PWH) in our study. This system assigned a confidence level ranging from 1 (highest confidence) to 4 (lowest confidence) based on various criteria. Level 1, indicating the highest confidence, included cases with an HIV viral load RNA quantification above 50 copies/mL at any point. Level 2 represented the next highest confidence, encompassing individuals who met at least two criteria from diagnosis, medication, or laboratory measures. Level 3 was assigned to cases with only medication evidence, and level 4, the lowest confidence, included individuals with exposure solely to ritonavir without meeting any other conditions or laboratory criteria. To minimize the risk of misclassifying HIV status in our analysis, we included only persons with HIV (PWH) classified at the higher confidence levels 1 and 2.

*Covariates (cont.)*

Below details ascertainment for variables descriptively summarized in Table 1 and Supplementary Table 2. Please note that those variables whose nonzero counts, after cross-classifying with key study factors such as race/ethnicity, are less than 20 have been suppressed both in counts and percentages. Further obfuscation is made of other counts in the same cross-classification by the following method: perturbing all but one of remaining counts, randomly selected, that share the same row total by an amount randomly selected from {-5,-4,-3,-2,-1,0,1,2,3,4,5} that maintains the same, fixed row total; percentages get recalculated accordingly.

Insurance Coverage: Insurance coverage information for individuals aged 19-64 was obtained from the American Community Survey data. This data was categorized into three levels – 'high,' 'medium,' and 'low.' These categories were established using tertile cut offs across the entire United States. The designation of 'high' indicates a high rate of health insurance coverage within the ZIP code, 'medium' represents an intermediate level, and 'low' signifies a low rate of health insurance coverage, specifically for people aged 19-64.[^1,2^](https://www.zotero.org/google-docs/?ZAiVVp)

Region: U.S. residency regions were classified into four categories – Northeast, Midwest, West, or South, based on the person's residential ZIP code[^3^](https://www.zotero.org/google-docs/?vHgjLC).

Body mass index (BMI): BMI was categorized into four groups: underweight (<18.5), healthy weight (≥18.5 and <25), overweight (25-30), and obese (≥30).[^4^](https://www.zotero.org/google-docs/?9k7NHU)

Number of COVID-19 vaccines received: The number of COVID-19 vaccines received by individuals was classified into four categories: 0, 1, 2, and 3 or more.

*Statistical analyses (cont.)*

*Stepwise modeling process.* Our stepwise modeling process utilized three main steps. Our first step modeled each SDoH factor, with a hierarchically-nested set of added variables for adjustments, for all individuals included in the analytic sample (Table 2). Our second step modeled each SDoH factor individually stratified by HIV status (Table 3). Our third, and final, step modeled all three SDoH factors, HIV status, and covariates stratified by race/ethnicity groups (Table 3). Below, we detail each stepwise modeling approach.

*Step 1- Modeling of each SDoH factor individually and COVID-19-related hospitalization for all individuals.* In this first step, we individually model each SDoH factor with the outcome, with first model entirely univariate (Model 1) and then incremental hierarchically-nested models adjusting for age, sex, and CCI (Model 2), HIV status (Model 3), and race/ethnicity (Model 4).

*Step 2- Modeling for each SDoH factor individually and COVID-19-related hospitalization stratified by HIV status.* In this second step, we conducted a univariate mixed effects logistic regression for each SDoH factor individually, stratified by HIV status, assuming a Gaussian-distributed random intercept across individual data partner sites.[^5^](https://www.zotero.org/google-docs/?SroOJU) This analysis aimed to explore the relationship between HIV status (as model stratification factor), each SDoH factor (as single predictors of outcome), and COVID-19-related hospitalization (outcome of interest); this in contrast to our later multivariable modeling, which was conducted stagewise. Our HIV-stratified “unadjusted” models still account for latent features that vary across data partner healthcare systems. By considering healthcare system-specific association estimates and accommodating heterogeneity within data systems, the analyses were effectively clustered by data partner site, providing nuanced insights into the conditional associations that are of direct interest to practicing clinicians and the patients they care for.

*Step 3- Modeling jointly for all SDoH factors and HIV status stratified by race/ethnicity.* Next, we conducted multivariable modeling analyses, stratified by race/ethnicity. Models were fit to each stratum’s data in a stagewise manner via both data-driven, minimally-adjusted models (denoted as “unadjusted” within each stratum) and fuller, clinical-risk-driven multivariable age-, sex-, and CCI-adjusted models (denoted “adjusted” within each stratum) to yield conditional-on-healthcare-system odds ratios for each SDoH factor and HIV status determined to be predictive of COVID-19 hospitalization via cross-validation. A number of other measured variables might have been considered, such as BMI and COVID-19 vaccination status, yet as such variables may well exhibit association in part due to their own association with upstream SDoH factors of primary interest – whose total effects constitute our primary estimands – we have not adjusted for these; see discussion in the epidemiologic methods literature[^6,7^](https://www.zotero.org/google-docs/?LSl0iL) for more on this rationale. Key exposures’ targeted total effects would appear attenuated in their regression coefficient estimates somewhat when (over-)adjusting for ‘intermediate’ variables, further supporting our rationale to exclude them along with others. To concretely confirm this latter issue during our investigations, we explored as *supplemental* analyses (ones targeting distinct, alternate association estimands), yielding results not shown that affirmed our rationale, as we next explain. It was most markedly confirmed by adjusting for an additional individual-level SDOH factor that falls outside the Healthy People 2030 domain areas, stress (excluded per rationale mentioned in the main paper). Notably, these items — posed to individuals in a time-limited way (see Epic wheel questions in Supplemental Table 1) such that it seems unclear how to resolve temporally relative to COVID-19 incidence via EHR records — it may have a mediating or moderating effect on both HIV status and targeted SDOH exposures (1-3) outlined above. Notably ‘adjustment’ may obscure true association effects of interest so, while we have not provided these supplemental estimates, we note this as a way to quantitatively explore this potential pitfall of overadjustment to readers of this report, should they endeavor to conduct similar analyses using other comparable data resources.

For all final multivariable models targeting our primary estimands, we chose to employ a data-driven approach to restricting the set of final covariates employed for adjustment within each stratum, primarily to mitigate the potential for overadjustment bias[^8^](https://www.zotero.org/google-docs/?qpxIdd) and, secondarily, due to concerns about certain strata’s limited amount of outcome information. Some covariate-induced substrata determined by a unique combination of covariate values, may face challenges due to sparse data, which hinders estimation (i.e., small or imbalanced expected cell counts for observed outcomes)[^9–11^](https://www.zotero.org/google-docs/?wo4ufH). Therefore, we restricted adjustments to those mean log-odds models consistent with a LASSO-regularized generalized linear model (GLM, binomial family). We chose the regularization parameter value that determined our final covariate set to be the optimal value selected via ten-fold cross-validation to yield performant discrimination between those with and without outcomes (i.e., per area under the receiver operating characteristic, or ROC, curve) (see, for example James, G., Witten, D., Hastie, T., Tibshirani, R. (2013). An Introduction to Statistical Learning: With Applications in R.) We chose this optimal-penalization approach in contrast with some practices, such as largest value within a single Monte Carlo standard error of the optimal value, that induce additional parsimony, since we do not entertain a large number of predictors and this strategy for increased parsimony (when studied via simulation with continuous outcomes) does not appear to outperform our chosen approach.[^12^](https://www.zotero.org/google-docs/?6JIOO5) An additional consideration may be the limited number of available data partner healthcare systems contributing to data heterogeneity for certain race/ethnicity strata (e.g., NH-AIAN), yet given the scale of N3C the smallest such number is 18 distinct healthcare systems, well within ranges in which GLMMs are deemed worthwhile (see, for example, Gelman & Hill 2007, Chapters 11 & 12)[^13^](https://www.zotero.org/google-docs/?Ougvbx). For the sake of visibility into each model’s provenance in terms of features selected as additive adjustment terms, we have reported the resulting estimates for each mean model’s GLM fit at the optimal regularization value, exhibiting values on both the log-odds (logit) scale as well as the odds ratio scale for direct comparison with final model fits, in Supplemental Table 5 below.

Due to large amount of missingness and our inability to assume missingness at random regarding the SDoH variables for the overall cohort, we proceeded with a subcohort for which one or more SDoH data were available, such that our results generalize to a population for which the individual was screened for one or more SDoH factors in the clinical setting and that data partner site included that data for ingestion in N3C. Our modeling approach accommodates missingness for all other variables (which is already shown descriptively in Table 1 and supplementary Table 2) as missing at random, by using all available data from the same healthcare system (from all instances whose data capture processes in their respective EHRs follow a shared workflow). Thus, assuming that, conditional on all other variables observed for this subcohort, any missing values within all other variables were modeled as missing at random by our likelihood-based framework of a mixed-effects generalized linear model (an available-case analysis, less restrictive in its tacit assumptions than a complete-case analysis; it is a rather subtle distinction to those less familiar with missing data methods literature yet mathematical subtleties are further explained in papers such as Thijs & al. 2002[^14^](https://www.zotero.org/google-docs/?hL5Eib) and Molenberghs & al., 1998[^15^](https://www.zotero.org/google-docs/?DTohmZ); additional subtleties on how some complete-case analyses can be sufficiently ‘adjusted’ while multiple imputation may be, in fact, biased – see, for example: Mathur & al., 2023[^16^](https://www.zotero.org/google-docs/?cgL2PB)). Such an approach as ours is akin to methods that employ multiple imputation while assuming that values are missing at random (see, for example, Gabrio & al., 2022[^17^](https://www.zotero.org/google-docs/?hFNiDU) and Siddique &al., 2011[^18^](https://www.zotero.org/google-docs/?u4LXYQ)). Thus, our overall approach uses available-case analysis for the SDoH data *at a data-contributing healthcare-system level* even if considered complete-case at a participant level, but our models functionally consider other missing data as missing at random.

Finally, we outline our rationale for using conditional-on-data-partners odds ratios. Our chosen method involves both an association effect measure that, while part of formal training for healthcare providers and associated with emerging standards like equalized odds as measures for fair/unbiased use of data important to racialized and marginalized communities, may not be universally employed (among research efforts reported in this journal) and, additionally, an extension to conventional regression methods that may not be familiar to all journal readers – employing mixed (fixed and random) effects. Recall that we have purposefully chosen estimands of association – odds ratios – that are familiar to a broad majority of clinical providers who constituted the largest part of decision making (admission to hospital at time of COVID-19 diagnosis) throughout the stages of the COVID-19 pandemic; schools of medicine, nursing, and allied health sciences have maintained odds ratios as a measure of association between two binary (dichotomous) variables within their quantitative curricula for decades, as well as within current examinations required for board certification[^19,20^](https://www.zotero.org/google-docs/?QVo0RQ), and despite recognized challenges in immediacy of interpretation by broader audiences, this choice was intentionally made to align with care providers’ expectations for quantifying disparities in outcomes like admission and mortality, including their expectations regarding a retrospective design like the current study. We look to take advantage of the odds ratio's symmetry, allowing for its interpretability across distinct types of prospective as well as retrospective designs, so this study's findings can inform the design of a greater variety of follow-on studies. Further, despite potential interpretability issues when generalizing, by being non-collapsible itself *and* being conditional on the referent log-odds of hospitalization for a given data partner healthcare system, collapsible measures of association (risk ratios) can be derived from model estimates (e.g., Hedeker & al., 2018[^21^](https://www.zotero.org/google-docs/?eA6BPA); Daniel & al.[^22^](https://www.zotero.org/google-docs/?0soLcM)); for example, should interested parties within a N3C-data-contributing partner system take interest, the predicted realization of their random referent log-odds could be useful in seeing how our findings could be placed in the context of their system.

**References**

[1. Bureau UC. Health Insurance Coverage in the United States: 2021. Census.gov. Accessed January 8, 2024. https://www.census.gov/library/publications/2022/demo/p60-278.html](https://www.zotero.org/google-docs/?ztiL1w)

[2. Bureau UC. American Community Survey Tables for Health Insurance Coverage. Census.gov. Accessed January 8, 2024. https://www.census.gov/data/tables/time-series/demo/health-insurance/acs-hi.html](https://www.zotero.org/google-docs/?ztiL1w)

[3. us_regdiv.pdf. Accessed January 26, 2024. https://www2.census.gov/geo/pdfs/maps-data/maps/reference/us_regdiv.pdf](https://www.zotero.org/google-docs/?ztiL1w)

[4. CDC. Assessing Your Weight. Centers for Disease Control and Prevention. Published June 9, 2023. Accessed January 26, 2024. https://www.cdc.gov/healthyweight/assessing/index.html](https://www.zotero.org/google-docs/?ztiL1w)

[5. A note on marginalization of regression parameters from mixed models of binary outcomes - Hedeker - 2018 - Biometrics - Wiley Online Library. Accessed January 5, 2024. https://onlinelibrary.wiley.com/doi/10.1111/biom.12707](https://www.zotero.org/google-docs/?ztiL1w)

[6. Rothman KJ, Greenland S, Lash TL. *Modern Epidemiology*. Third edition. Wolters Kluwer Health/Lippincott Williams & Wilkins; 2008. Accessed January 5, 2024. http://www.r2library.com/public/ResourceDetail.aspx?authCheck=true&resid=2127](https://www.zotero.org/google-docs/?ztiL1w)

[7. Explanation in Causal Inference. Accessed January 7, 2024. https://global.oup.com/us/companion.websites/9780199325870/](https://www.zotero.org/google-docs/?ztiL1w)

[8. Schisterman EF, Cole SR, Platt RW. Overadjustment Bias and Unnecessary Adjustment in Epidemiologic Studies. *Epidemiology*. 2009;20(4):488. doi:10.1097/EDE.0b013e3181a819a1](https://www.zotero.org/google-docs/?ztiL1w)

[9. Lesaffre E, Albert A. Partial Separation in Logistic Discrimination. *Journal of the Royal Statistical Society Series B (Methodological)*. 1989;51(1):109-116.](https://www.zotero.org/google-docs/?ztiL1w)

[10. Mehta CR, Patel N, Senchaudhuri P. Exact Stratified Linear Rank Tests for Ordered Categorical and Binary Data. *Journal of Computational and Graphical Statistics*. 1992;1(1):21-40. doi:10.2307/1390598](https://www.zotero.org/google-docs/?ztiL1w)

[11. Hirji KF, Mehta CR, Patel NR. Computing Distributions for Exact Logistic Regression. *Journal of the American Statistical Association*. 1987;82(400):1110-1117. doi:10.2307/2289388](https://www.zotero.org/google-docs/?ztiL1w)

[12. Chen Y, Yang Y. The One Standard Error Rule for Model Selection: Does It Work? *Stats*. 2021;4(4):868-892. doi:10.3390/stats4040051](https://www.zotero.org/google-docs/?ztiL1w)

[13. Gelman A, Hill J. Data Analysis Using Regression and Multilevel/Hierarchical Models. Higher Education from Cambridge University Press. doi:10.1017/CBO9780511790942](https://www.zotero.org/google-docs/?ztiL1w)

[14. Thijs H, Molenberghs G, Michiels B, Verbeke G, Curran D. Strategies to fit pattern‐mixture models. *Biostatistics*. 2002;3(2):245-265. doi:10.1093/biostatistics/3.2.245](https://www.zotero.org/google-docs/?ztiL1w)

[15. Monotone missing data and pattern‐mixture models - Molenberghs - 1998 - Statistica Neerlandica - Wiley Online Library. Accessed April 12, 2024. https://onlinelibrary.wiley.com/doi/abs/10.1111/1467-9574.00075](https://www.zotero.org/google-docs/?ztiL1w)

[16. Mathur M, Shpitser I, VanderWeele TJ. A common-cause principle for eliminating selection bias in causal estimands through covariate adjustment. Published online January 31, 2023. doi:10.31219/osf.io/ths4e](https://www.zotero.org/google-docs/?ztiL1w)

[17. Linear mixed models to handle missing at random data in trial‐based economic evaluations - Gabrio - 2022 - Health Economics - Wiley Online Library. Accessed April 12, 2024. https://onlinelibrary.wiley.com/doi/full/10.1002/hec.4510](https://www.zotero.org/google-docs/?ztiL1w)

[18. Siddiqui O. MMRM versus MI in Dealing with Missing Data—A Comparison Based on 25 NDA Data Sets. *Journal of Biopharmaceutical Statistics*. 2011;21(3):423-436. doi:10.1080/10543401003777995](https://www.zotero.org/google-docs/?ztiL1w)

[19. NCLEX & Other Exams. NCSBN. Accessed February 2, 2024. https://www.ncsbn.org/nclex.page](https://www.zotero.org/google-docs/?ztiL1w)

[20. Corrigan B. USMLE Content Outline.](https://www.zotero.org/google-docs/?ztiL1w)

[21. Hedeker D, du Toit SHC, Demirtas H, Gibbons RD. A Note on Marginalization of Regression Parameters from Mixed Models of Binary Outcomes. *Biometrics*. 2018;74(1):354-361. doi:10.1111/biom.12707](https://www.zotero.org/google-docs/?ztiL1w)

[22. Making apples from oranges: Comparing noncollapsible effect estimators and their standard errors after adjustment for different covariate sets - Daniel - 2021 - Biometrical Journal - Wiley Online Library. Accessed February 2, 2024. https://onlinelibrary.wiley.com/doi/10.1002/bimj.201900297](https://www.zotero.org/google-docs/?ztiL1w)

## **Supplementary Tables & Figures**

**Supplementary Table 1. Individual-level SDoH domains assessed via Epic Wheel questions and responses with mapping to Healthy People 2030 categorization and study metadata**

| **Epic Wheel category** | **Healthy People 2030 domain** | **Question** | **Responses** | **Indicates need, risk or instability** |
| --- | --- | --- | --- | --- |
| Housing | Economic stability | Are you worried about losing your housing [PRAPARE] | I choose not to answer this question | Unknown |
|  |  |  | Negative | No |
|  |  |  | Positive | Yes |
|  |  |  | No | No |
|  |  |  | Yes | Yes |
|  |  | Housing status | I have housing | No |
|  |  |  | I do not have housing (staying with others, in a hotel, in a shelter, living outside on the street, on a beach, in a car, or in a park) | Yes |
|  |  |  | At risk | Yes |
|  |  |  | I have a place to live today, but I am worried about losing it in the future | Yes |
|  |  |  | No matching concept | Unknown |
| Social connectedness | Social cohesion | Marital status [NHANES] | Married | No |
|  |  |  | Never married | No |
|  |  |  | Divorced | Yes |
|  |  |  | Refused | Unknown |
|  |  |  | Widowed | Yes |
|  |  |  | Living with partner | No |
|  |  |  | Separated | Yes |
|  |  |  | No matching concept | Unknown |
|  |  |  | Single | No |
|  |  |  | Living as married | No |
|  |  |  | Don't know | Unknown |
|  |  | Do you belong to any clubs or organizations such as church groups unions, fraternal or athletic groups, or school groups [NHANES III] | No | Yes |
|  |  |  | Yes | No |
|  |  |  | No matching concept | Unknown |
|  |  |  | Patient refused | Unknown |
|  |  |  | Not asked | Unknown |
| Stress | NA | Do you feel stress - tense, restless, nervous, or anxious, or unable to sleep at night because your mind is troubled all the time - these days [OSQ] | Not at all | No |
|  |  |  | Only a little | No |
|  |  |  | To some extent | Yes |
|  |  |  | Rather much | Yes |
|  |  |  | Very much | Yes |
|  |  |  | No matching concept | Unknown |
|  |  |  | Patient refused | Unknown |
|  |  |  | Not asked | Unknown |
| Transportation | Access to health services | Has lack of transportation kept you from medical appointments, meetings, work, or from getting things needed for daily living | No | No |
|  |  |  | I choose not to answer this question | Unknown |
|  |  |  | Yes, it has kept me from medical appointments or from getting my medications | Yes |
|  |  |  | Yes, it has kept me from non-medical meetings, appointments, work, or from getting things that I need | Yes |
|  |  |  | Yes | Yes |
|  |  |  | No matching concept | Unknown |
|  |  |  | Patient refused | Unknown |
|  |  |  | Not asked | Unknown |
| Financial strain | Economic stability | How hard is it for you to pay for the very basics like food, housing, medical care, and heating | Not hard at all | No |
|  |  |  | Not very hard | No |
|  |  |  | Somewhat hard | Yes |
|  |  |  | Hard | Yes |
|  |  |  | Very hard | Yes |
|  |  |  | No matching concept | Unknown |
|  |  |  | Hardly at all | No |
|  |  |  | Patient refused | Unknown |
|  |  |  | Not asked | Unknown |
|  |  |  | No | No |
|  |  |  | Yes | Yes |
|  |  |  | Already shut off | Yes |
| Food insecurity | Economic stability | Within the past 12 months we worried whether our food would run out before we got money to buy more [U.S. FSS] | Never true | No |
|  |  |  | Sometimes true | Yes |
|  |  |  | DK or Refused | Unknown |
|  |  |  | Often true | Unknown |
|  |  |  | No matching concept | Unknown |
|  |  |  | Patient refused | Unknown |
|  |  |  | Not asked | Unknown |
|  |  |  | Don't know/refused | Unknown |
|  |  |  | Never true | No |
|  |  |  | Sometimes true | Yes |
|  |  |  | DK or Refused | Unknown |
|  |  |  | Often true | Yes |
|  |  |  | Patient refused | Unknown |
|  |  |  | No matching concept | Unknown |
|  |  |  | Not asked | Unknown |
|  |  |  | Don't know/refused | Unknown |
| Abbreviations: PRAPARE= Protocol for Responding to and Assessing Patients’ Assets, Risks , and Experiences,  NHANES= National Health And Nutrition Examination Survey,  OSQ= The Occupational Support Questionnaire,  FSS= Family Self Sufficiency program | | | | |

**Supplementary Table 2: Baseline characteristics of people living with HIV (PWH) and without HIV (PWOH) among COVID-19 positive individuals included in analysis in the U.S. N3C, January 2020- November 2023 (N=280,441)**

| **Variable Category** | **Category level** | **PWH [n=3291, N=16 data partner sites]** | **PWOH [n=277150, N=24 data partner sites]** | **Overall [n=280,441, N=24 data partner sites]** |
| --- | --- | --- | --- | --- |
| **Race/ethnicity** | NH- American Indian or Alaska Native (AIAN) | <20 (*) | 1599 (0.57) | 1608 (0.57) |
|  | NH- Asian American, Native Hawaiian, or Pacific Islander (AANHPI) | 99 (3.01) | 5940 (2.14) | 6039 (2.15) |
|  | NH- Black or African American | 760 (23.09) | 37358 (13.47) | 38118 (13.59) |
|  | Hispanic/Latinx of any race | 212 (6.44) | 21778 (7.85) | 21990 (7.84) |
|  | NH- White | 2211 (67.18) | 210475 (75.94) | 212686 (75.84) |
| **Sex** | Male | 1495 (54.57) | 170841 (61.64) | 107804 (38.44) |
|  | Female | 1796 (45.42) | 106309 (38.35) | 172637 (61.55) |
| **Age** | 18-44 | 1198 (36.40) | 84563 (30.51) | 85761 (30.58) |
|  | 45-64 | 732 (22.24) | 111005 (40.05) | 111737 (39.84) |
|  | >65 | 1361 (41.35) | 81582 (29.44) | 82943 (29.57) |
| **Insurance coverage by zip code for individuals aged 19-64^1^** | High (>93.1) | 1258 (38.22) | 89141 (32.16) | 90399 (32.23) |
|  | Medium (<93.1, >86.0) | 1204 (36.58) | 95955 (34.62) | 97159 (34.64) |
|  | Low (<86.0) | 828 (25.16) | 71770 (25.90) | 72598 (25.89) |
|  | Null | <20 (*) | 20284 (7.32) | 20285 (7.23) |
| **Region of participant resident** | Northeast | 63 (1.91) | 18048 (6.51) | 18111 (6.46) |
|  | Midwest | 1683 (51.14) | 123447 (44.54) | 125130 (44.62) |
|  | South | 1267 (38.50) | 104036 (37.54) | 105303 (37.55) |
|  | West | 278 (8.45) | 20020 (7.22) | 20298 (7.24) |
|  | Missing | - | 11599 (4.18) | 11599 (4.13) |
| **CCI score, median (IQR)** |  | 2 (4,0) | 1 (4,0) | 1 (4,0) |
| **BMI^2^** | Underweight | <20 (*^1^) | 1062 (0.38) | 1078 (0.38) |
|  | Healthy weight | 333 (10.12) | 31757 (11.46) | 32090 (11.44) |
|  | Overweight | 829 (25.19) | 68784 (24.81) | 69613 (24.82) |
|  | Obese | 2104 (63.93) | 160841 (58.03) | 162945 (58.10) |
|  | Missing | <20 (*^1^) | 14706 (5.31) | 14715 (5.25) |
| **Number of COVID-19 vaccinations** | 0 | 1244 (37.80) | 132802 (47.92) | 134046 (47.80) |
|  | 1 | 369 (11.21) | 22248 (8.03) | 22617 (8.06) |
|  | 2 | 507 (15.40) | 44415 (16.02) | 44922 (16.02) |
|  | 3 or more | 1171 (35.58) | 77685 (28.03) | 78856 (28.12) |
| **Access to healthcare issue** | Yes | 146 (4.44) | 5306 (1.91) | 5452 (1.94) |
|  | No | 2828 (85.93) | 205915 (74.30) | 208743 (74.43) |
|  | Missing | 317 (9.63) | 65929 (23.79) | 66246 (23.62) |
| **Social cohesion issue** | Yes | 1752 (53.23) | 107580 (31.82) | 109332 (38.98) |
|  | No | 973 (29.57) | 83442 (30.11) | 84415 (30.10) |
|  | Missing | 566 (17.20) | 86128 (31.08) | 86694 (30.91) |
| **Economic stability issue** | Yes | 662 (20.11) | 28091 (10.13) | 28753 (10.25) |
|  | No | 2473 (75.14) | 213402 (77.00) | 215875 (76.98) |
|  | Missing | 156 (4.74) | 35657 (12.86) | 35813 (12.78) |
| **COVID-19 related hospitalization** | Yes | 302 (9.18) | 31208 (11.26) | 31510 (11.23) |
| Abbreviations: NH=Non-Hispanic/Latinx, CCI= Charlson Comorbidity Index  ^*^This cell count is associated with a nonzero count, that is <20. Thus, to align with N3C agreements we do not populate the corresponding proportion (%) of the value and we obfuscate° the remaining cell counts in the row to prevent any meaningful back-calculation of this nonzero but <20 cell count.  ^1^Insurance: Insurance coverage data for individuals aged 19-64, reflecting 'high,' 'medium,' and 'low' categories, was sourced from the American Community Survey. These classifications, based on nationwide tertile cutoffs, denote varying rates of health insurance coverage within the zip code  CCI: derived from binary flags for comorbidities before the first incident COVID-19 infection, excluding HIV  ^2^BMI categories are: underweight (<18.5), healthy weight (≥18.5 and <25), overweight (25-30), and obese (≥30)  ° For more detail on the obfuscation method employed with contingent cell counts, see supplement. | | | | |

**Supplementary Table 3: Results for covariate estimates from modeling each individual-level SDoH factor and COVID-19-related hospitalization stratified by HIV status in the U.S. N3C, January 2020- November 2023 (N=280,441)**

| **SDoH factor model** | **Covariate** | **PWH (n=3291)**  **aOR (95% CI)** | **PWOH (n=277150)**  **aOR (95% CI)** |
| --- | --- | --- | --- |
| Access to healthcare issue | Age, years  <45  45-64  65+ | Ref.  0.88 (0.62, 1.25)  0.81 (0.54, 1.23) | Ref.  1.03 (0.99, 1.08)  **1.42 (1.36, 1.48)** |
|  | Male sex (female referent) | **1.37 (1.03, 1.85)** | **1.30 (1.26, 1.34)** |
|  | Charlson comorbidity index (CCI) | **1.21 (1.15, 1.26)** | **1.22 (1.21, 1.23)** |
| Economic stability issue | Age, years  <45  45-64  65+ | Ref.  0.91 (0.65, 1.29)  0.99 (0.66, 1.47) | Ref.  **1.07 (1.03, 1.12)**  **1.48 (1.42, 1.54)** |
|  | Male sex (female referent) | 1.23 (0.93, 1.62) | **1.32 (1.28, 1.36)** |
|  | Charlson comorbidity index (CCI) | **1.21 (1.16, 1.26)** | **1.21 (1.20, 1.21)** |
| Social cohesion issue | Age, years  <45  45-64  65+ | Ref.  0.77 (0.51, 1.17)  0.89 (0.55, 1.43) | Ref.  1.03 (0.97, 1.09)  **1.51 (1.43, 1.60)** |
|  | Male sex (female referent) | 1.19 (0.85, 1.65) | **1.22 (1.18, 1.27)** |
|  | Charlson comorbidity index (CCI) | **1.24 (1.18, 1.30)** | **1.27 (1.26, 1.28)** |
| Abbreviations: PWH=people living with HIV; PWOH=people living without HIV; OR=odds ratio; CI=confidence interval  **Bold text** indicates estimates with *p*-values <0.05  ^1^Notes: Unadjusted odds ratios obtained via modeling each SDoH factor (as exposure) and COVID-19-related hospitalization (as outcome) using mixed-effects logistic regression, with random effects restricted to a random intercept, i.e., referent log-odds, for each unique data partner  ^2^Adjusted odds ratios obtained via modeling each SDoH factor (as exposure) and COVID-19-related hospitalization (as outcome) using mixed-effects logistic regression, with random effects restricted to a random intercept, i.e., referent log-odds, for each unique data partner, and adding regression terms for age (categorical), sex, and Charlson Comorbidity Index (CCI, continuous) | | | |

**Supplementary Table 4: Results for covariate estimates from modeling jointly individual-level SDoH factors, HIV status, and COVID-19-related hospitalization stratified by race/ethnicity in the U.S. N3C, January 2020- November 2023 (N=280,441)**

| **Covariate** | **NH-American Indian or Alaska Native (N=1608)** | **NH-AANHPI (N=6039)** | **NH-Black/African American (N=38118)** | **Hispanic/Latinx  of any race (N=21990)** | **NH-White (N=212686)** |
| --- | --- | --- | --- | --- | --- |
|  | **Adjusted estimates (95% CI)** | **Adjusted estimates (95% CI)** | **Adjusted estimates (95% CI)** | **Adjusted estimates (95% CI)** | **Adjusted estimates (95% CI)** |
| **Age >65** | 1.48 (0.96, 2.30) | **2.03 (1.52, 2.71)** | 1.48(1.37, 1.60) | **2.27 (2.05, 2.51)** | **1.91 (1.81, 2.01)** |
| **Age 45-64** | 1.27 (0.83, 1.95) | 1.03 (0.77, 1.42) | 1.01 (0.94, 1.09) | **1.21 (1.09, 1.34)** | **1.19 (1.13, 1.26)** |
| **Male: Female** | **1.72 (1.26, 2.35)** | 0.90 (0.72, 1.13) | **1.48 (1.40, 1.57)** | **1.51 (1.40, 1.63)** | **1.27 (1.23, 1.31)** |
| **CCI** | **1.20 (1.15, 1.26)** | **1.29 (1.25, 1.34)** | **1.19 (1.18, 1.20)** | **1.13 (1.11, 1.14)** | **1.22 (1.21, 1.22)** |
| Abbreviations: NH=Non-Hispanic/Latinx; AANHPI=Asian American, Native Hawaiian, or Pacific Islander  Primary Multivariable analysis results by racial/ethnic groups: Data Partner Site Conditional* Odds Ratios (and 95% confidence intervals) estimated via GLMM-fitted models (random intercepts by data partner site); mean model features selected per GLM-cross-validation-specified regularization**  * “conditional” in the sense that all odds ratio estimates are interpreted as *conditional on* the referent log-odds of hospitalization predicted for each data partner healthcare system, as modeling approach to accommodate heterogeneity across systems  **mean models selected via cross-validation per LASSO (L1 regularized) fits at minimum (optimal) lambda value… then maintaining age-/sex-/Charlson-Comorbidity-Index variables for *further* ‘adjusted’ estimates; “_”=covariate not selected. | | | | | |

**Supplementary Table 5: Intermediate Multivariable Regression Coefficient Estimates from penalized regression (naȉve* generalized linear model for binomial family) determined by cross-validation to select covariates for adjustment in final mixed-effects models**

|  | **NH-American Indian or Alaska Native**  **(N=1,608)** | | **NH-AANHPI**  **(N= 6,039)** | | **NH-Black/African American**  **(N= 38,118)** | | **Hispanic/Latinx  of any race**  **(N=21,990)** | | **NH-White**  **(N=212,686)** | |
| --- | --- | --- | --- | --- | --- | --- | --- | --- | --- | --- |
|  | **Unadjusted estimate (95% CI)** | **Adjusted estimates (95% CI)** | **Unadjusted estimate (95% CI)** | **Adjusted estimates (95% CI)** | **Unadjusted estimate (95% CI)** | **Adjusted estimates (95% CI)** | **Unadjusted estimate (95% CI)** | **Adjusted estimates (95% CI)** | **Unadjusted estimate (95% CI)** | **Adjusted estimates (95% CI)** |
| **Referent odds (probability)** | -1.685 (0.156) | -2.564 (0.0715) | -2.5523 (0.0723) | -3.4602 (0.0305) | -1.283(0.217) | -2.251(0.0953) | -1.3696 (0.2027) | -2.0677 (0.1123) | -2.1547 (0.1039) | -3.2688 (0.0367) |
| *Log-odds ratio scale* |  | | | |  | | | | | |
| **HIV positive** | _ | _ | _ | _ | 0.072_ | _ | -0.2674 | _ | -0.4830 | -0.2142 |
|  |  |  |  |  | -0.258 | -0.006 |  |  |  |  |
| **Social cohesion issue** | -0.6272 | -0.2929 | -0.318 | -0.1463 | -0.3895 | -0.3416 | -0.8452 | -0.5812 | -0.4316 | -0.3118 |
| **Economic instability** | 0.1326 | -0.0303 | 0.4261 | 0.1631 | -0.0745 | -0.0303 | _ | _ | 0.1256 | 0.1558 |
| **Access to health services issue** | 0.2512 | 0.2189 | 0.6917 | 0.5319 | 0.2986 | 0.2189 | 0.0423 | _ | 0.7650 | 0.5884 |
| **Male** | _ | 0.3358 | _ | -0.0218 | _ | 0.3720 | _ | 0.2567 | _ | 0.1739 |
| **Age >65** | _ | 0.3352 | _ | 0.6317 | _ | 0.3352 | _ | 0.6589 | _ | 0.5135 |
| **Age 45-64** | _ | _ | _ | _ | _ | _ | _ | _ | _ | _ |
| **Charlson Comorbidity Index** | _ | 0.1797 | _ | 0.2610 | _ | 0.1797 | _ | 0.1237 | _ | 0.2103 |
| *Odds ratio scale* |  | | | |  | | | | | |
| **HIV positive** | 1.075 | _ | _ | _ | 1.075_ | _ | 0.7654 | _ | 0.6169 | 0.8072 |
| **Social cohesion issue** | 0.5340 | 0.7461 | 0.7271 | 0.8639 | 0.6774 | 0.7106 | 0.4295 | 0.5592 | 0.6494 | 0.7322 |
| **Economic instability** | 1.1418 | 0.9701 | 1.5312 | 1.1771 | 0.9282 | 0.9701 | _ | _ | 1.1338 | 1.1686 |
| **Access to health services issue** | 1.2856 | 1.245 | 1.9970 | 1.7021 | 1.348 | 1.245 | 1.0432 | _ | 2.1491 | 1.8011 |
| **Male** | _ | 1.399 | _ | 0.9784 | _ | 1.451 | _ | 1.2926 | _ | 1.1900 |
| **Age >65** | _ | 1.398 | _ | 1.881 | _ | 1.398 | _ | 1.9326 | _ | 1.6711 |
| **Age 45-64** | _ | _ | _ | _ | _ | _ | _ | _ | _ | _ |
| **Charlson Comorbidity Index** | _ | 1.197 | _ | 1.2982 | _ | 1.197 | _ | 1.1316 | _ | 1.2340 |
| *****estimates provided without measures of uncertainty (standard errors / confidence intervals) as they are naive with respect to inherent data partner healthcare system heterogeneity in outcome-predictor relationships; provided merely as provenance to predictor selection; “_” denotes term NOT selected for mean model LASSO (L1 regularized) fit optimized via cross-validation for outcome discrimination. | | | | | | | | | | |
